# Single-scan rest/stress imaging with ^99m^Tc-Sestamibi and cadmium zinc telluride-based SPECT for hyperemic flow quantification: A feasibility study evaluated with cardiac magnetic resonance imaging

**DOI:** 10.1371/journal.pone.0183402

**Published:** 2017-08-17

**Authors:** Yu-Hua Dean Fang, Yuan-Chang Liu, Kung-Chu Ho, Feng-Cheng Kuo, Ching-Fang Yang, Tzu-Chen Yen, I-Chang Hsieh

**Affiliations:** 1 Department of Biomedical Engineering, National Cheng Kung University, Tainan, Taiwan; 2 Department of Medical Imaging and Intervention, Chang Gung Memorial Hospital, Linkou, Taiwan; 3 Department of Nuclear Medicine, Chang Gung Memorial Hospital, Linkou, Taiwan; 4 Department of Medical Imaging and Radiological Sciences, Chang Gung University, Taoyuan, Taiwan; 5 Center for Advanced Molecular Imaging and Translation & Cyclotron Center, Chang Gung Memorial Hospital, Linkou, Taiwan; 6 Department of Cardiology, Chang Gung Memorial Hospital, Linkou, Taiwan; University of Alabama at Birmingham, UNITED STATES

## Abstract

**Introduction:**

We aimed to evaluate whether the hyperemic myocardial blood flow (MBF) can be estimated using cadmium zinc telluride (CZT)-based single-photon emission computed tomography (SPECT) cameras with a single, rapid rest/stress dynamic scan. Dynamic contrast-enhanced (DCE) cardiac magnetic resonance imaging (MRI) was used as a reference modality for flow measurement.

**Materials and methods:**

The proposed protocol included both the rest and stress acquisitions within a 24-min scan. Patients were first injected with 99mTc-Sestamibi at the resting state. Sixty minutes after the first injection, the subject was positioned via scintigraphy, after which the list-mode data acquisition was initiated and continued for 24 minutes. Five minutes after data acquisition was initiated, a stressed state was induced via dipyridamole infusion, after which a second dose of 99mTc-Sestamibi was injected. Dynamic SPECT images were reconstructed for all subjects, who also underwent T1-weighted cardiac DCE-MRI performed on days other than those of the SPECT studies. MBF values were estimated for the rest and stress MRI studies, and for the stress portion of the SPECT study. The SPECT-measured hyperemic MBF was compared with the MR-measured hyperemic MBF and coronary flow reserve (CFR), based on the regions of interest.

**Results:**

A total of 30 subjects were included in this study. The hyperemic MBF estimated from SPECT showed a strong correlation with the MR-measured hyperemic MBF (r^2^ = 0.76) and a modest correlation with the MR-measured CFR (r^2^ = 0.56). Using MR-measured CFR <1.3 as a cutoff for coronary stenosis, we found that the SPECT-measured hyperemic MBF served as a useful clinical index with 94% sensitivity, 90% specificity, and 93% accuracy.

**Conclusions:**

Hyperemic MBF can be measured with a rapid, single-scan rest/stress study with CZT-based SPECT cameras.

## Introduction

Flow quantification based on non-invasive myocardial perfusion imaging (MPI) has long been a much-desired capability in the clinical evaluation of coronary artery disease (CAD). The quantitative assessment of myocardial blood flow (MBF) and coronary flow reserve (CFR) has gradually become part of the routine analysis for cardiac PET [1–3] and magnetic resonance imaging (MRI) [4, 5]. Various reports have shown that the image-based measurement of MBF and CFR helps physicians achieve more precise diagnosis of CAD [1, 5–7], particularly patients suspected of having multi-vessel disease [3, 8]. Novel SPECT scanners are equipped with cadmium zinc telluride (CZT) detectors that yield a high detection sensitivity and have a stationary detector design [9]; several reports have shown that these scanners enable rapid dynamic acquisitions for absolute MBF and CFR quantification [8, 10–14]. Current data also indicate that SPECT-based quantification is generally accurate and strongly correlated with PET-based measurements [13], fractional flow reserve (FFR) [12], and invasive coronary angiography (CAG) [8, 12, 14]. Although the methodology may still require further refinement, standardization, and validation, flow quantification using the CZT cameras is strongly expected to become a routine procedure in the near future [10, 15–17]. Given the advantages of SPECT MPI in terms of cost and popularity, considerable efforts have been made to transform the CZT-based SPECT MPI into a completely quantitative method for flow measurement.

Despite the promise this technology holds, there are several practical issues that need to be addressed before SPECT-based MBF quantification can be routinely used. In particular, the customization of the workflow design for quantitative SPECT MPI remains a priority, due to various reasons. First, to quantify the MBF, the kinetic modeling approach typically requires the immediate initiation of dynamic acquisition with bolus tracer injection or slightly before the injection. A dynamic scan would require an on-table injection, which would prolong the scan due to patient preparation. As a result, the entire rest/stress scan would require approximately 40–50 minutes of scanner time for each patient. Thus, this becomes a time-demanding task that devalues the speed advantage offered by highly sensitive CZT cameras. The other practical challenge is the small field of view (FOV) of CZT-based cameras dedicated for cardiac applications. Such cameras have a reduced FOV to improve the spatial resolution of reconstructed images under a fixed detector gantry [9, 18]. For conventional rest/stress protocols, the tracer injection is performed off-table. Therefore, technicians can appropriately adjust the patient position and place the heart in the center of the FOV before the scan begins, via real-time scintigraphy. However, when the bolus injection occurs on the table and the acquisition starts immediately with the injection, adjustment of the position based on pre-existing activity is no longer possible for the first acquisition. This FOV issue represents another challenge of dynamic rest/stress SPECT MPI. Consequently, a customized design of the scanning protocol is essential to achieve flow quantification, without sacrificing patient throughput or image quality for CZT-based cameras dedicated for MPI.

To address these challenges, we propose a new single-scan rest/stress protocol and related data processing methods in the present study. Our aim was to evaluate whether the hyperemic MBF can be accurately estimated using CZT-based SPECT cameras with a rapid, single rest/stress dynamic scan. In our protocol, the patient is first injected with tracer, off the table, under resting conditions. After allowing for some uptake time, the patient is scanned dynamically using a CZT camera. The initial part serves as acquisition at rest. During the on-the-table continuous dynamic acquisition, the patient is pharmaceutically stressed and then administered another dose of tracer. Dynamic scanning is continued throughout the period of pharmaceutical stress. Thus, both the rest and stress portions will be acquired with a single scan. The dynamic time activity curves facilitate kinetic modeling analysis and quantification of the hyperemic MBF. Our working hypothesis is that the hyperemic MBF can be properly estimated with this protocol and provide similar diagnostic value as the CFR. To evaluate the accuracy of the SPECT-estimated hyperemic MBF from this single-scan rest/stress SPECT study, cardiac rest/stress MRI studies were used as the reference of comparison in a clinical trial with human subjects. If the hyperemic MBF can be measured accurately by CZT-based SPECT with our rapid, single-scan rest/stress protocol, CZT-based SPECT could provide a practical approach to quantify the hyperemic MBF in routine MPI examinations.

## Materials and methods

### Acquisition protocol design

**Fig 1:**
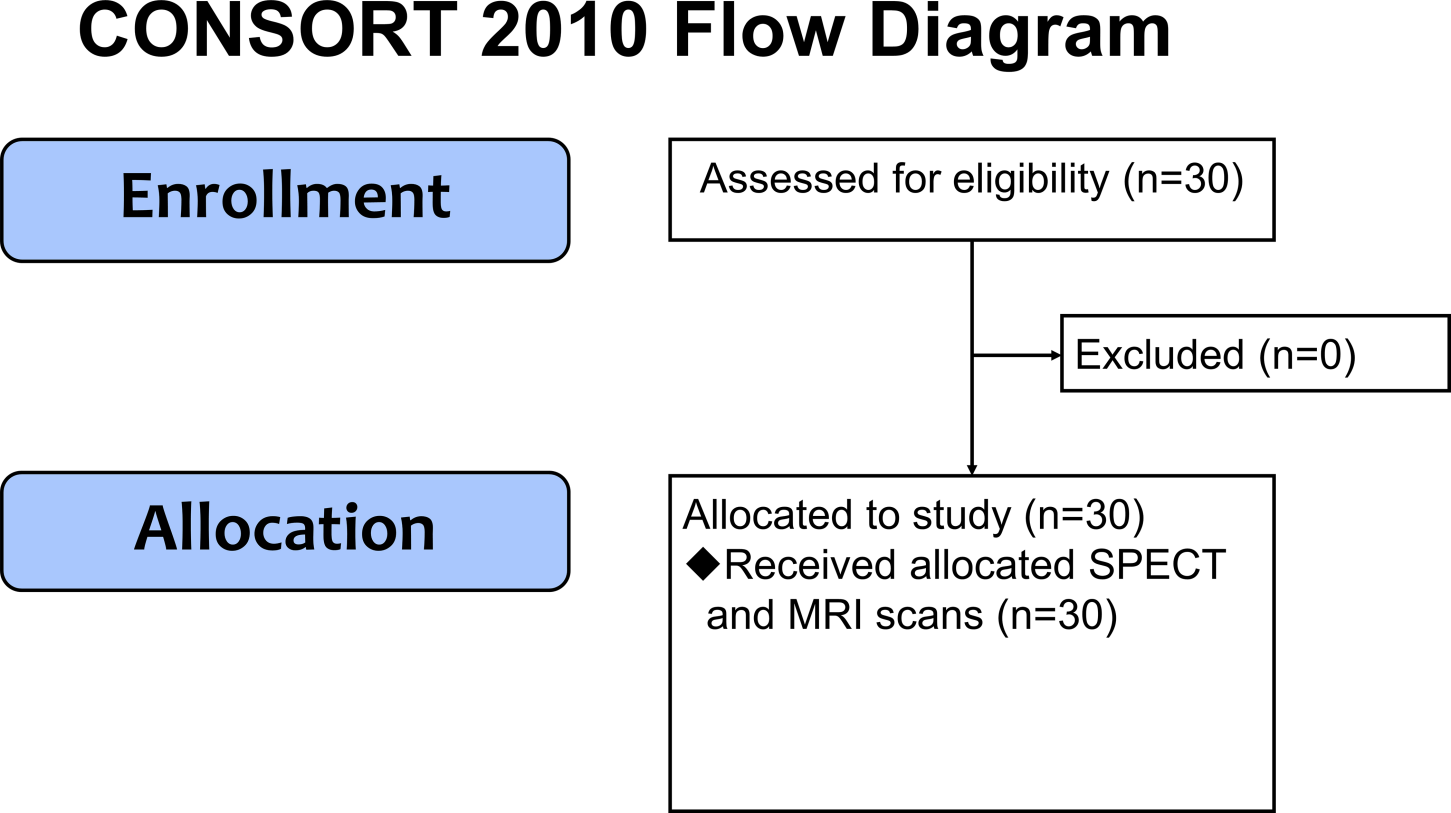
CONSORT flow diagram of the clinical trial.

**Fig 2:**
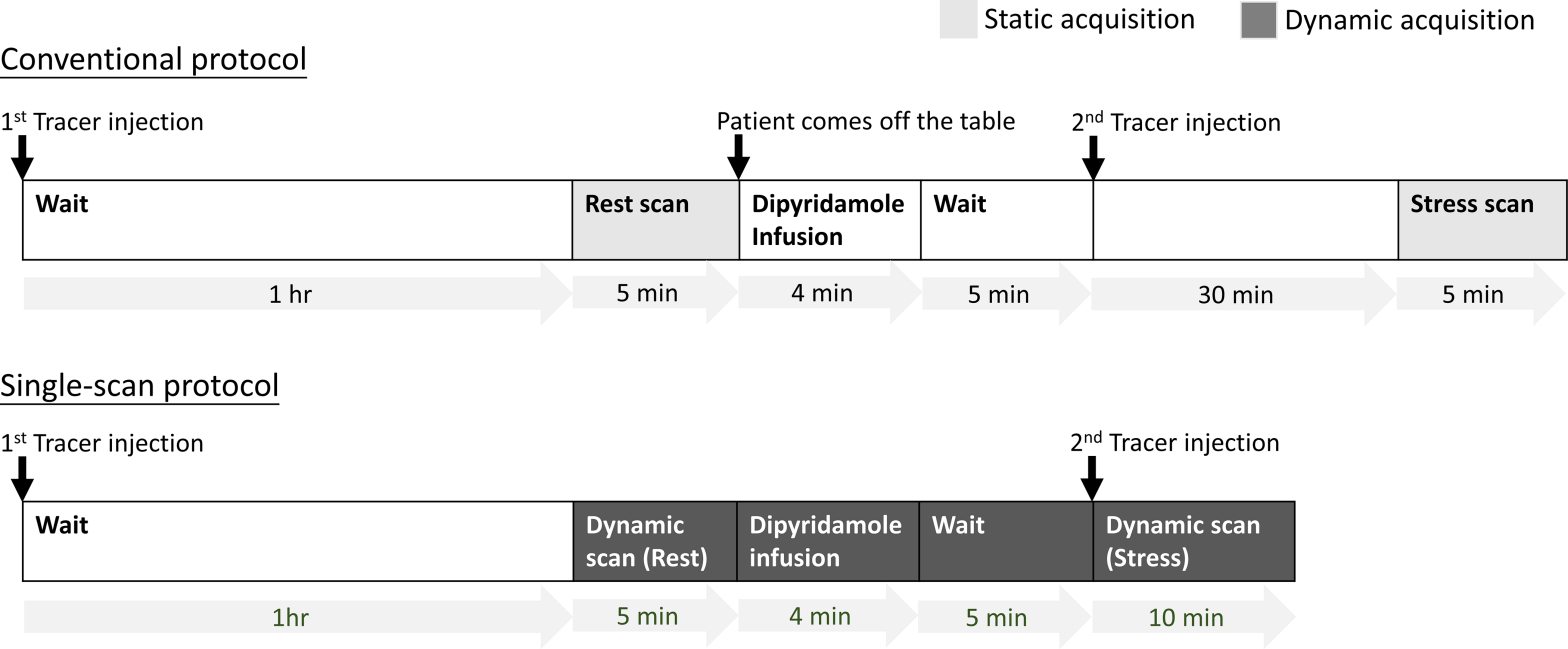
Comparison between the conventional and proposed protocols.

The CONSORT flow diagram of the clinical trial is shown in Fig 1. The proposed scan protocol was illustrated in Fig 2. A subject under study was first injected with 10 mCi of ^99m^Tc-Sestamibi under resting conditions as a bolus intravenous injection. After permitting uptake for 60 minutes, the subject was placed on the scanner table and a technologist carefully adjusted the patient position via scintigraphy such that the heart was placed at the center FOV. Once the patient was appropriately positioned, data acquisition was initiated in list-mode and continued for 24 minutes. After 5 minutes of scanning, the subject was pharmaceutically stressed with 0.142 mg/kg/min of dipyridamole that was slowly infused over 4 minutes. Five minutes after the end of dipyridamole infusion, 20 mCi of ^99m^Tc-Sestamibi was intravenously injected as a bolus. The scan was terminated 10 minutes after the second tracer injection. Thus, the whole acquisition can be separated into three phases: rest acquisition (5 min), transition between the resting and stress states (9 min), and stress acquisition (10 min). The third part (i.e., the stress state) involves the summation of the activity from the stress state and the residual activities from the previous phase.

### Hyperemic flow quantification with SPECT

To simplify data processing, the time-activity curve (TAC) obtained from a cardiac region of interest was separated into two parts. The first 5 minutes of the TAC included the activity from purely the resting state. By assuming that the residual activity during the transition state remained approximately constant (as in the resting state), the activity of the last 10 minutes could be considered a summation of the residual activity during the resting state and the activity following stress and the second tracer injection. To exclude the effect of residual activity in the resting state from the stress portion, the mean activity of the first 5 minutes was calculated and then subtracted from the whole TAC.

To obtain the input function required for kinetic modeling analysis, a region of interest (ROI) was drawn in the left ventricular (LV) cavity over a short-axis slice. In this slice, another ROI was drawn over the myocardium. Correction for the partial volume effect and spillover of the LV TAC was performed using the model-corrected input function method [17]. The resultant input function was a fitted version described by a 7-parameter equation [19]. A hematocrit value of 0.45 was used to convert the whole-blood concentration to the plasma concentration. The residual activity of the rest acquisition was subtracted from that of the stress acquisition. Once the tissue stress TACs were determined for the LV and myocardium, the tissue TACs were fitted with a two-tissue-compartment model using the LV TACs as input functions with the COMKAT software [7, 20, 21]. The estimated kinetic rate constant K_1_ was used to approximate the hyperemic MBF. The proposed protocol and the data analysis procedures were deposit in protocols.io and can be found at https://dx.doi.org/10.17504/protocols.io.iqjcdun.

### Clinical data acquisition

In order to evaluate the accuracy of the estimated hyperemic blood flow using SPECT MPI, we conducted a prospective clinical trial. The trial was approved by the institutional review board (IRB) of Chang Gung Memorial Hospital, Linkou. All the pharmaceuticals used in this study, including dipyridamole, ^99m^Tc-Sestamibi, Gd-DTPA and aminophylline, were covered under the study protocol approved by the IRB. The inclusion and exclusion criteria were listed in S1 File. All the subjects signed the informed consent form approved by the IRB. A total of 30 subjects were recruited in this study between May 2012 and May 2015. Their information was summarized in Table 1. All subjects were suspected as having coronary artery disease based on the symptoms of chest pain, apnea, shortness of breath, or previous cardiac events. Once a subject consented to participate in the trial, the subject would undergo both a SPECT MPI and a cardiac MRI exam with a ≤2-week interval; only one subject underwent the examinations with a 20-day interval due to scheduling issues. All subjects were fasted for at least 4 hours before both the SPECT and MRI exams. After the SPECT and MRI exams were conducted, an experienced nuclear physician interpreted the SPECT images and an experienced radiologist interpreted the MR images. Based on their findings, a cardiologist then evaluated whether the subject should receive invasive CAG. All the involved physicians were board certified. Among the 30 subjects, four were scheduled to receive further CAG; all these four subjects had coronary stenosis >60% in least one of the three major coronary branches.

**Table 1:** General characteristics of the study patients (n = 30).

Characteristic	Data
**Demographics**	
Age (years)	63.5 ± 8.3
Female sex	11 (36.7%)
Hypertension	22 (73.3%)
Height (cm)	162.6 ± 8.2
Weight (kg)	71.4 ± 11.6
**Coronary risk factors**	
Dyslipidemia	19 (63.3%)
Diabetes	6 (20%)
Familial history of CAD	4 (13.3%)
Tobacco use	8 (26.7%)
**Medical history**	
Prior PCI	17 (56.7%)
Prior stenting	16 (53.3%)
**Symptoms**	
Chest pain	15 (50%)
Angina	17 (56.7%)
Dyspnea	3 (10%)

The study was retrospectively registered at ClinicalTrials.gov because the investigators were not aware of the requirement of trial registration. ClinicalTrials.gov registration number is NCT02985931. The authors confirm that all ongoing and related trials for this study are registered.

Using the proposed SPECT acquisition protocol, all subjects underwent image acquisition with a CZT-based SPECT camera (Discovery NM 530c, GE Healthcare). This camera has a multiple pinhole collimator and 19 fixed-angle CZT detectors [22]. After a dynamic acquisition, images were reconstructed into 48 30-second frames using built-in software (Myovation for Alcyon, GE Healthcare). The reconstruction was performed with the 3-D iterative Bayesian reconstruction algorithm [23, 24] into a 70-by-70 matrix with 50 slices. The pixel size was set as 4 mm in all three directions. Scatter correction was not performed, although the high energy resolution of the CZT-based camera provided good scatter rejection, as reported previously [24].

The MRI acquisitions followed a standard cardiac MR stress/rest protocol [7, 25]. For each subject, the dynamic contrast-enhanced (DCE) MR image was acquired on the short axis over the basal, mid, and apical slices, with an additional long-axis view of the LV. The pixel size was set at 1.875 mm. Each subject was slowly infused with 0.142 mg/kg/min dipyridamole for 4 minutes. After 5 minutes, the DCE MR image was acquired with a T1-weighted turboFLASH sequence (TR/TE/TI = 152/1/90 milliseconds). Ten seconds after the scan was initiated, 0.2 mL/kg Gd-DTPA (Magnevist, Bayer Pharma AG) was given as a bolus injection. DCE-MRI scanning was continued for 90 seconds. After the stress study was completed, the antidote aminophylline was administered as a bolus injection. Approximately 20 minutes later, another DCE-MRI scan was acquired with the subject in the resting state.

### Flow measurement obtained via SPECT and MRI

We evaluated the flow measurement obtained via SPECT and MRI on a ROI basis. For each subject, two ROIs were drawn in an individually selected slice. For the subjects with CAG-confirmed coronary stenosis (n = 4), an ROI was placed over the corresponding stenosis site and the other ROI was placed at the contralateral location in the myocardium. For those who did not undergo CAG (n = 26), the ROIs were placed in the anterior and posterior locations of the mid short-axis MR slice. All ROIs were manually drawn over the MR images and then duplicated to the corresponding short-axis SPECT images using in-house software. The ROI location on the SPECT images was carefully checked to match the ROI coverage between the MR and SPECT images. For the SPECT data, TAC were generated from the dynamic images and fitted to the model using the data processing procedures described in the previous section of hyperemic flow quantification with SPECT. The estimated K_1_ in the stress portion of the scan was considered as the hyperemic MBF. For the MRI data, the time-intensity curves were converted into Gd-DTPA concentrations assuming that the Gd-DTPA concentration is proportional to the relative post-contrast intensity change normalized to the pre-contrast intensities [26–29]. Then a one-compartment model was used to fit the time-concentration curve for estimating the kinetic rate constants. The K_trans_ for all ROIs during the rest and stress studies were estimated and considered as the MR-measured baseline and hyperemic MBF values. The CFR was defined as the ratio between the stress and rest MBF values, measured using MRI. All data analysis was performed using MATLAB (version R2015a, Mathworks Inc. Natick, Massachusetts).

### Statistical analysis

From each ROI, four parameters were estimated: the SPECT-measured hyperemic MBF, the MR-measured CFR, baseline MBF values, and hyperemic MBF values. The correlation between the hyperemic MBF values measured using MR and SPECT was assessed using regression analysis and Pearson’s correlation coefficient. We also performed regression analysis to evaluate the correlation between the SPECT-measured MBF and the MR-measured CFR. Bland–Altman plots were used to evaluate the agreement between SPECT-measured MBF and the MR-measured MBF as well as the MR-measured CFR. In addition to regression analysis, we also wished to investigate the diagnostic value of the SPECT-measured hyperemic MBF. Accordingly, we used the criteria of MR-measured CFR <1.3 to indicate coronary ischemia [4]. Receiver operating characteristic (ROC) analysis was performed for SPECT-measured hyperemic MBF against the reference based on MR-measured CFR. The area under the ROC curve (AUC) was determined, along with the optimal accuracy, sensitivity, and specificity for SPECT-measured MBF.

## Results

**Fig 3:**
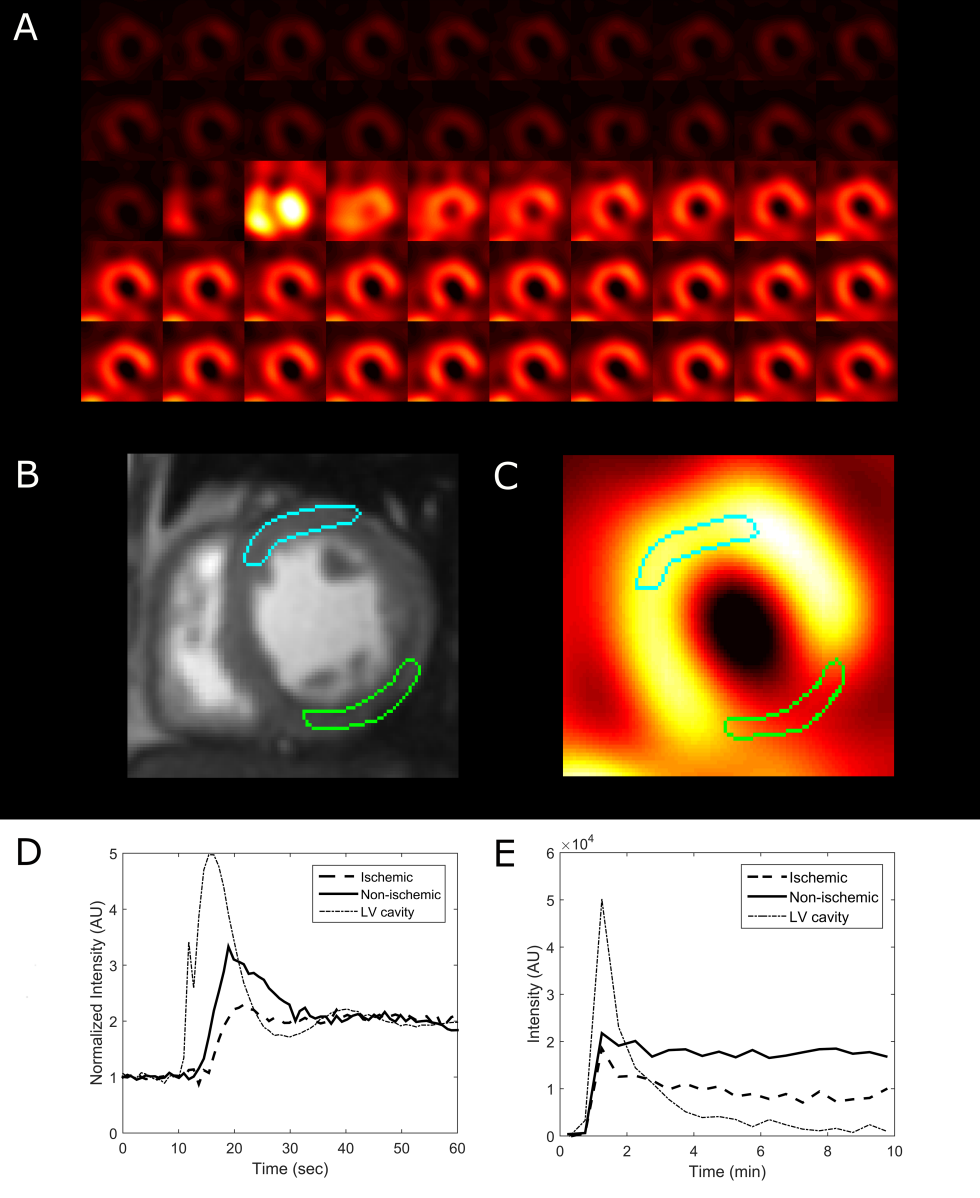
A representative case for flow quantification in a 70-year-old woman. (A) Dynamic SPECT images of a short-axis slice. During the second injection, the bright spots represent the left and right ventricular cavities. (B) The MR slice and ROI placement over the ischemic (green) and non-ischemic (blue) regions. (C) ROI placement of the SPECT image. (D) Time-intensity curves of the LV cavity, and ischemic and non-ischemic ROIs derived via MRI. (E) Time-intensity curves of the LV cavity, and ischemic and non-ischemic ROIs derived via SPECT. In the ischemic region, the hyperemic MBF was found to be 0.73 using MRI and 0.82 using SPECT. In the non-ischemic region, the hyperemic MBF was found to be 2.06 using MRI and 1.65 using SPECT. CAG indicated a 92% stenosis in the mid-LCX.

**Fig 4:**
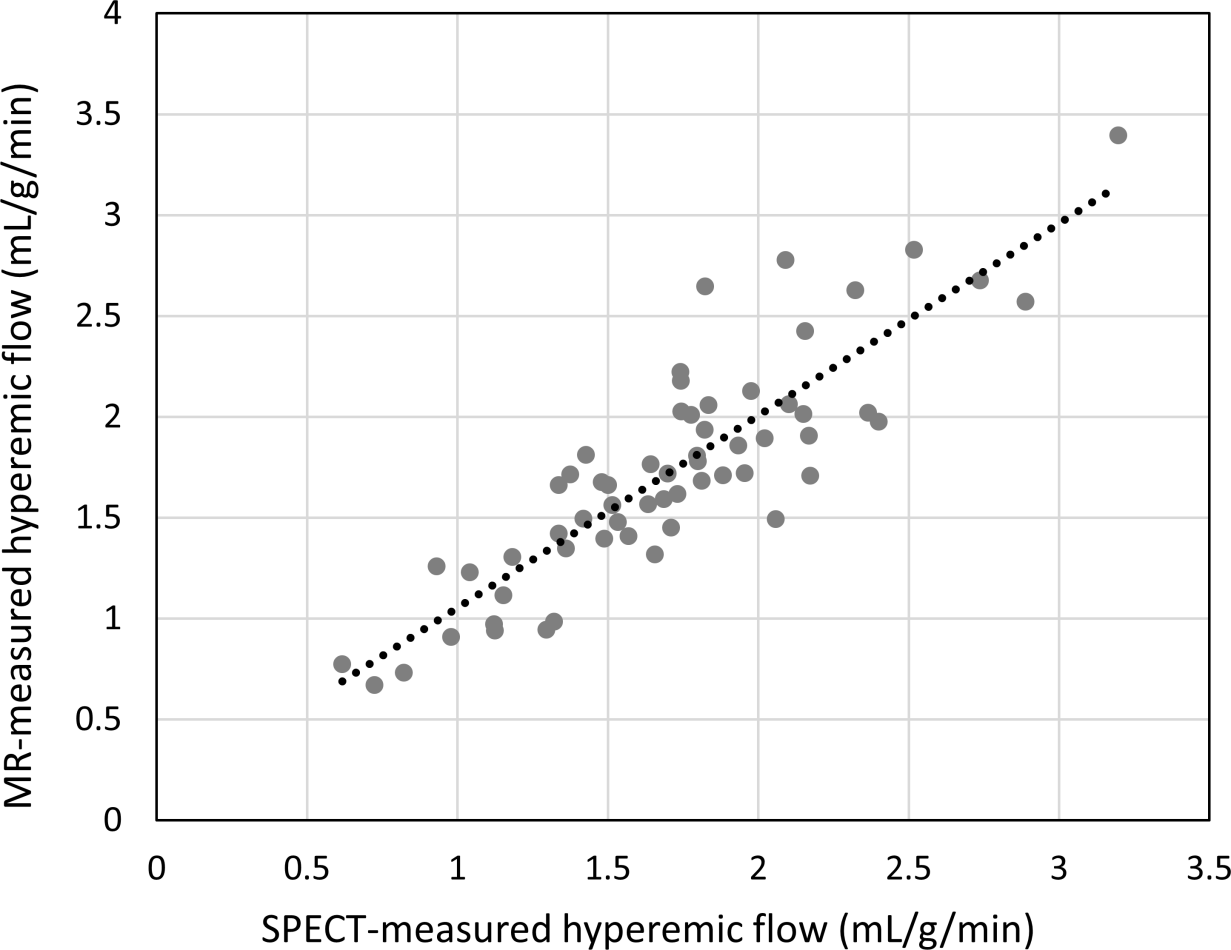
Scatter diagram and regression line of SPECT-measured hyperemic MBF *versus* MR-measured hyperemic MBF. Pearson’s correlation coefficient r^2^ = 0.76. The slope was 0.95.

**Fig 5:**
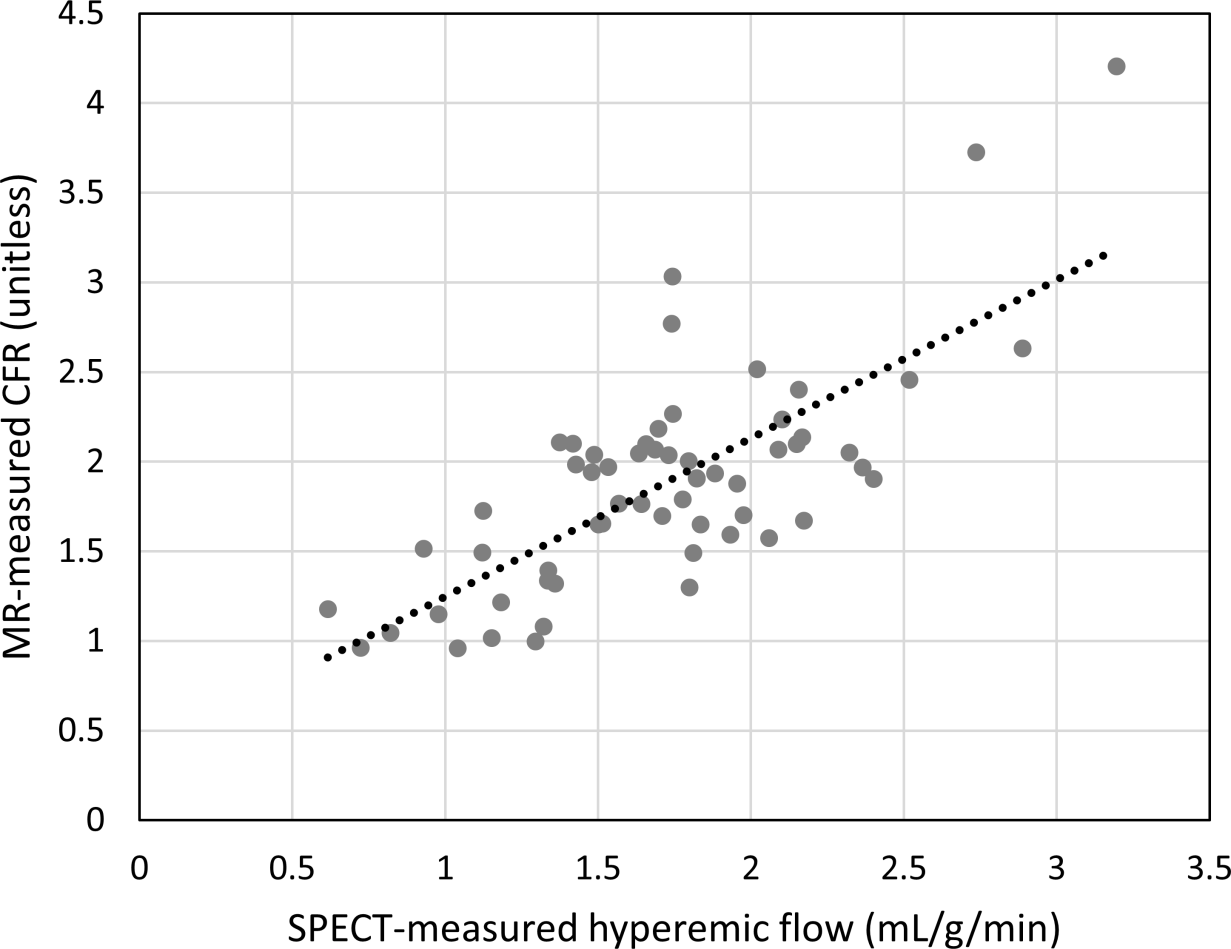
Scatter plot of SPECT-measured hyperemic MBF versus MR-measured CFR. Pearson’s correlation coefficient r^2^ = 0.56. The slope was 0.88.

**Fig 6:**
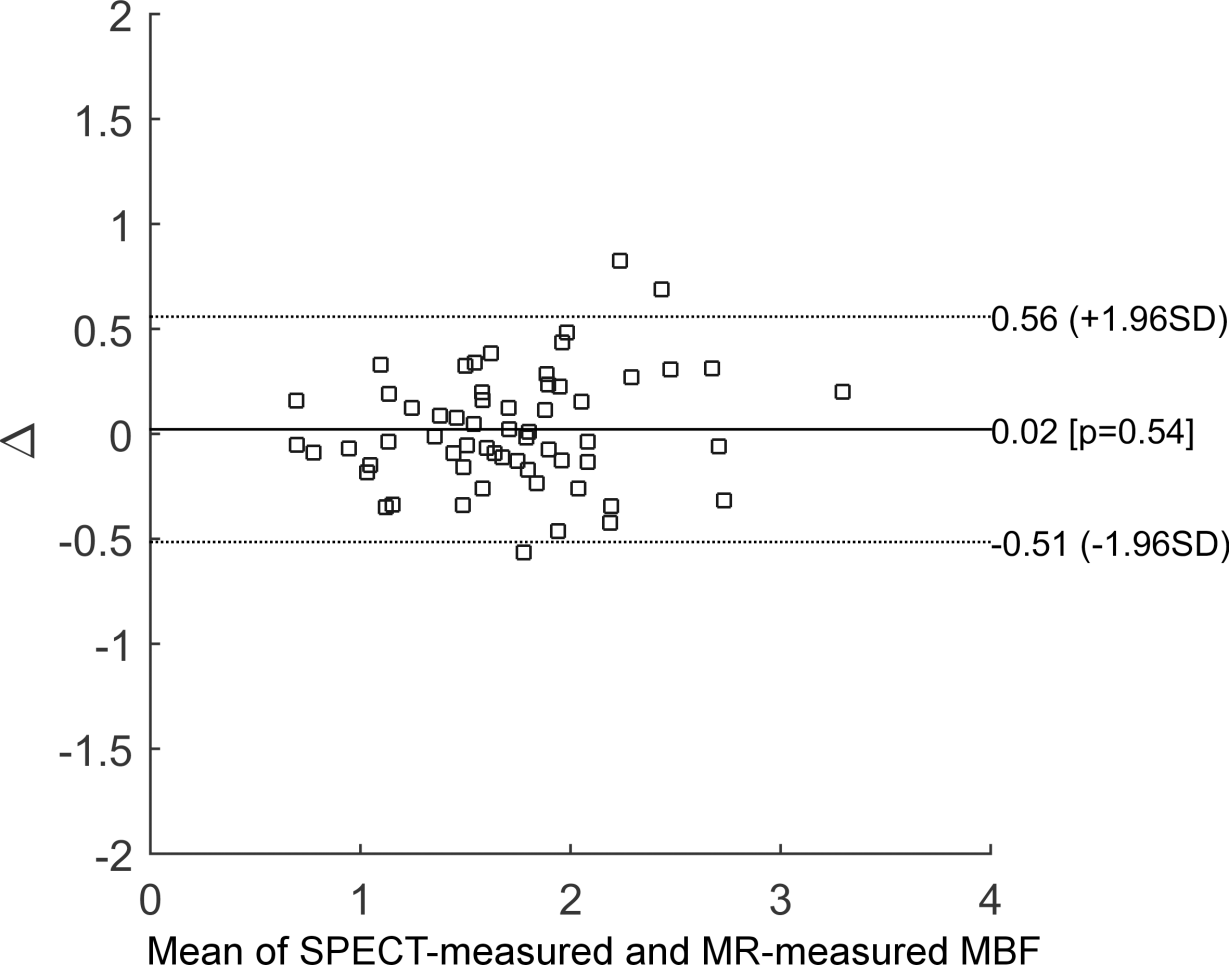
Bland–Altman plot of SPECT-measured hyperemic MBF *versus* MR-measured hyperemic MBF. 95% of the data points fell within the limits of agreement, indicating good agreement between the MBF measured with these two methods.

**Fig 7:**
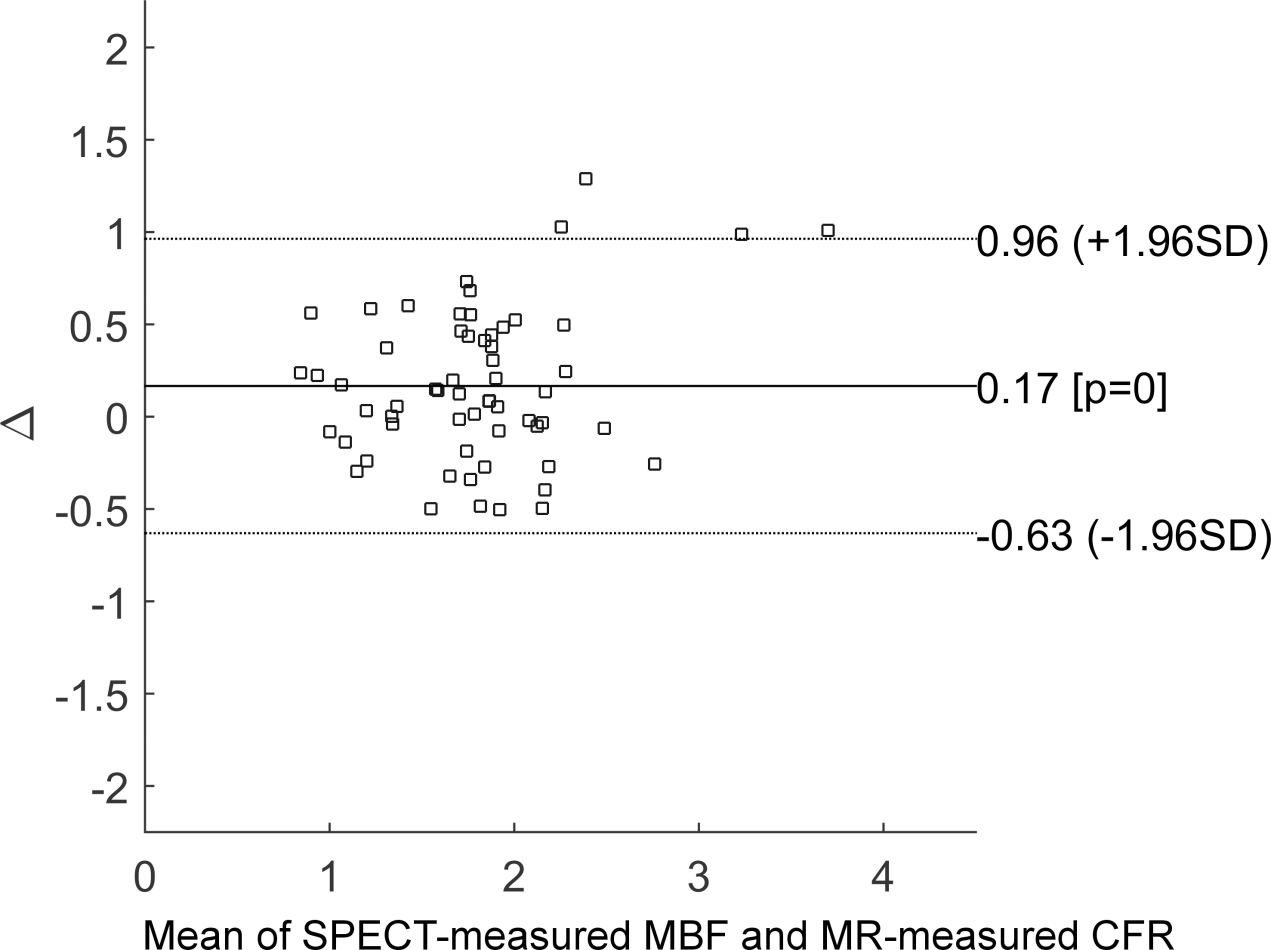
Bland–plot of SPECT-measured hyperemic MBF *versus* MR-measured CFR. 93% of the data points fell within the limits of agreement, indicating modest agreement between the SPECT-measured hyperemic MBF and CFR.

**Fig 8:**
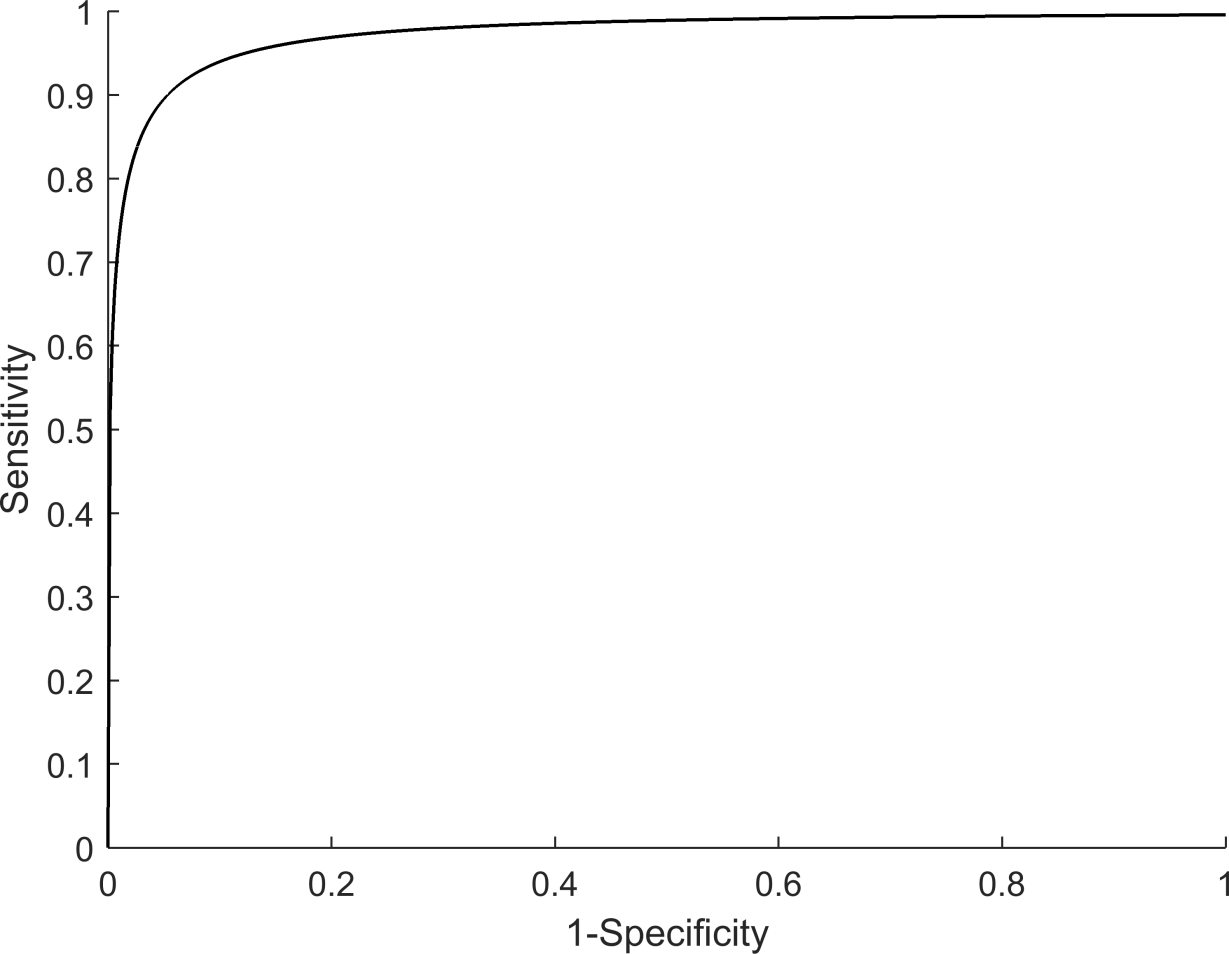
Resultant curve from the ROC analysis of the SPECT-measured hyperemic MBF. The AUC was found to be 0.90.

Hyperemic MBF values were estimated for all 30 subjects with SPECT, using the proposed protocol, and the described data analysis procedures. The dynamic images and TACs of one representative subject are illustrated in Fig 3. The statistics of the image quantification results are summarized in Table 2. In brief, in the four subjects who underwent PCI exams that confirmed coronary stenosis, the SPECT-measured hyperemic MBF was 0.85 ± 0.20 mL/g/min^-1^ in the ischemic regions and 2.01 ± 0.34 mL/g/min^-1^ in the non-ischemic regions. In the remaining subjects, the SPECT-measured hyperemic MBF was 1.77 ± 0.46 mL/g/min^-1^. Using cardiac MRI, the subjects who underwent CAG were estimated to have a hyperemic MBF of 0.81 ± 0.12 mL/g/min^-1^ in the ischemic regions and 1.94 ± 0.13 mL/g/min^-1^ in the non-ischemic regions. The remaining subjects were found to have an MR-estimated hyperemic MBF of 1.73 ± 0.56 mL/g/min^-1^. In the regression analysis of the hyperemic MBF estimated using SPECT and MR, Pearson’s correlation coefficient (r^2^) was found to be 0.76, when using the best fitting line of MBF_MR_ = 0.9522 × MBF_SPECT_ + 0.1033, as shown in Fig 4. When comparing the SPECT-measured hyperemic MBF and the MR-measured CFR, the correlation coefficient r^2^ was found to be 0.56, when using the best fitting line of CFR_MR_ = 0.8839 × MBF_SPECT_ + 0.3645, as shown in Fig 5. The Bland–Altman plot was shown in Fig 6 for the SPECT-measured and MR-measured hyperemic MBF, whereas another plot was shown in Fig 7 for the SPECT-measured hyperemic MBF and MR-measured CFR. Using the CFR<1.3 criteria [4], the ROC analysis of the SPECT-measured hyperemic MBF showed an overall accuracy, sensitivity, and specificity of 93%, 94%, and 90%, respectively. The AUC was found to be 0.90. The ROC curve was shown in Fig 8. The optimal cutoff of the SPECT-measured hyperemic MBF was 1.32 mL/g/min^-1^.

**Table 2:** Results of statistical analysis of the MBF and CFR estimated using MR and SPECT.

	CFR_MR_	Hyperemic MBF_MR_	Hyperemic MBF_SPECT_
All subjects	1.87 ± 0.60	1.73 ± 0.56	1.71 ± 0.51
CAG subjects (ischemic regions)	1.21 ± 0.30	0.81 ± 0.12	0.85 ± 0.20
CAG subjects (non-ischemic regions)	1.62 ± 0.30	1.94 ± 0.14	2.01 ± 0.34
Non-CAG subjects	1.95 ± 0.60	1.80 ± 0.51	1.77 ± 0.46

MBF units: ml/g/min

CFR: dimensionless

The supplemental file S2 File contains additional graphs to illustrate the curve fitting details, the gender-specific statistical graphs, and the Bland-Altman plots of the CAG and non-CAG groups.

## Discussion

With the emergence of novel tools that allow physicians to examine the heart in multiple aspects in terms of anatomy and function, the diagnosis and clinical management of coronary stenosis have rapidly evolved. Instead of simply observing the degree of arterial narrowing, physicians can now consider and integrate multiple functional parameters to achieve maximal benefit for the patient [30]. Among those parameters, MBF and the derived CFR have been widely used for evaluating ischemic severity. PET has played a significant role in quantifying baseline and hyperemic MBF. With fast-decaying PET tracers such as ^15^O-water and ^82^Rb, the rest and stress flow values can be efficiently measured within a single short scan. PET tracers with longer half-lives have also found to facilitate a single rest/stress scan [31, 32]. In general, SPECT has the advantage of an easier access to radiopharmaceuticals because SPECT perfusion tracers have longer half-lives than PET perfusion tracers. This advantage, combined with the relatively lower cost of scanners and tracers, makes SPECT a more commonly adopted modality for cardiac MPI in nuclear medicine. In terms of flow quantification, SPECT has evolved gradually as compared to PET. Flow quantification with CZT-based SPECT cameras has been widely explored in recent years. Novel SPECT scanners are equipped with cadmium zinc telluride (CZT) detectors that, compared to PMT-based scanners, yield higher detection sensitivity, better count rate capacity, improved spatial resolution, superior energy resolution, enhanced contrast-to-noise, and have a stationary detector design [9] that obviates gantry rotation and facilitates dynamic scanning with short frames. All these advantages are valuable for absolute MBF and CFR quantification with SPECT [8, 10–14]. Fast and high-SNR dynamic scans enable myocardial flow quantification with SPECT MPI. Several studies have demonstrated the feasibility, accuracy, and usefulness of such quantification [8, 12, 13], but only few efforts have focused on the design of a customized workflow for SPECT-based quantitative MPI studies. Due to the limited FOV and long half-lives of SPECT perfusion tracers, the current protocols have some limitations and consequently represent a barrier for the routine use of quantitative SPECT MPI. For example, a single scan protocol was adopted by Bouallègue et al. [12] to complete rest/stress study within approximately 21 minutes. In order to appropriately position the heart, this protocol actually requires the use of three tracer injections. Furthermore, since the pharmaceutical stress was started shortly after the first tracer injection (~6 minutes), the residual activity from the first acquisition is actually affected by the vasodilators, thus adding complexity to data processing [32]. The use of separate scan protocols may reduce issues related to fluctuation [8]. However, such protocols would be time-consuming as patients will need to be prepared separately for rest and stress acquisitions. The difference in patient position may also lead to a difference in the attenuation path for the two acquisitions. Moreover, the use of three tracer injections to appropriately position the patient for the first acquisition, is unavoidable. We believe that these practical issues hinder the adoption of protocols that enable SPECT-based flow quantification in a busy nuclear cardiology department. Hence, we sought to establish a novel and customized protocol that would achieve a good balance between patient throughput and quantitative ability.

The protocol proposed herein is a single-scan rest/stress protocol that enables the quantification of hyperemic MBF. With a 24-minute acquisition period and prior patient positioning, this protocol can be completed within 30 minutes. Moreover, it requires only two injections as with conventional static scans, instead of three injections. With the single scan design, the patient position remains fixed between the rest and stress acquisitions. The disadvantage includes the lack of flow quantification at the resting state, which does not permit the quantification of CFR. We believe that this is a necessary sacrifice, considering the additional time and injection demands of resting MBF quantification. Furthermore, some recent reports imply that hyperemic MBF alone may be sufficient to evaluate ischemic severity. For instance, Danad et al. reported that hyperemic flow measured with ^15^O-water PET provided similar diagnostic performances, as with CFR, for CAD detection [33, 34]. Another recent report by Stuijfzand et al. even showed that hyperemic MBF slightly outperformed CFR [35]. Although additional data are required to determine whether hyperemic MBF can actually serve as a clinical surrogate of CFR, we decided to design our protocol as a tradeoff of quantitative capability to the timing efficiency. This protocol maintains the hyperemic MBF quantification. Moreover, it remains to be a rest/stress study, as the rest acquisition still enables conventional visual interpretation. The final benefit of such a design is that the pharmacological stress is initiated at a much later stage, as compared to the first tracer injection, and will therefore have a minimum effect on the residual activity from the first tracer injection. This constant residual activity will simplify modeling analysis by allowing us to subtract the residual activity from the stress TAC.

Based on our data, the proposed protocol appears to be satisfactory for measuring the hyperemic MBF with CZT-based SPECT. Several data processing techniques have been used for the quantification procedures, including the model-corrected input functions, residual subtraction for stress TAC, and compartment modeling. The SPECT-estimated hyperemic flow values were similar to those measured with MR in all subgroups. In regression analysis, we observed a strong correlation between SPECT- and MR-measured hyperemic MBF (r^2^ = 0.76). There was only a slight over-estimation in the SPECT-measured flow, with a slope of 0.95. Bland–Altman plot analysis showed that SPECT-measured MBF was in good agreement with the MR-measured one. These data indicated the feasibility of the proposed protocol and analysis procedures for achieving a reliable estimation of the hyperemic MBF with CZT-based SPECT cameras. In contrast, when comparing SPECT-measured hyperemic MBF and CFR, the correlation of SPECT-measured hyperemic MBF versus CFR (r^2^ = 0.56) was less satisfactory. However, when using the MR-measured CFR cutoff of 1.3 as a diagnostic criterion, the SPECT-measured hyperemic flow provides a reliable discrimination of ischemic ROIs, with high sensitivity (94%) and high specificity (90%). This suggests that although the correlation between hyperemic flow and CFR is modest, the diagnostic value of the hyperemic flow remains satisfactory. Such a finding is consistent with previous efforts to compare the diagnostic performance of hyperemic MBF and CFR [33–37], thus implying that hyperemic MBF estimation may suffice for routine evaluation when CFR measurement is not feasible for practical reasons.

The main goal of this report is to demonstrate the feasibility of a rapid single-scan rest/stress protocol and the accuracy of SPECT-based hyperemic MBF quantification under the proposed protocol. Nevertheless, some improvements can be made to our proposed protocol. In particular, several strategies can be used to further reduce the time required. For example, the uptake time of 60 minutes may be further reduced to ≤30 minutes, as reported in the literature [38, 39]. Moreover, the acquisition time for the stress portions could be shortened to approximately 6 minutes [8]. If a fast-acting vessel dilator such as regadenoson is used, the waiting time to reach peak stress can also be reduced to <1 minute [38, 40]. By combining all these time-saving strategies, we expect that a single-scan rest/stress dynamic study can be completed within 15–20 minutes. Such a protocol would provide about the same patient throughput as the conventional static rest/stress protocol does, offering the additional capability of hyperemic MBF quantification. As the main purpose of this report was feasibility evaluation, only a small population was included; hence, we could not test all the possible strategies for shortening the scan time. For further verification and validation, a large cohort is required, in order to evaluate the accuracy, reproducibility, and clinical benefits. Efforts should also be made to establish standardized data analysis algorithms, preferably to automate these processes in the future. The data analysis components, including input function determination, kinetic model configuration, and residual activity handling, should all be carefully evaluated and optimized before SPECT-based MBF quantification becomes a clinical routine.

Yet another improvement would be to initiate the dynamic scan at the time of the injection of the first tracer dose. This improvement would allow the dynamic scan to capture the time-activity curves of the first minutes of the resting state, thus facilitating the quantification of the SPECT-measured resting MBF. Combined with the hyperemic MBF, the modified protocol would enable the measurement of CFR. However, such an improvement is not possible using the current scanner because the pre-existing tracer activity is required to position the patient’s heart in the FOV using scintigraphic data before the initiation of the acquisition. If a large FOV can be used to ensure proper positioning even without pre-existing tracer activity, or if positioning can be done with CT on a SPECT/CT scanner, then modifying the current protocol to enable the measurement of resting MBF is surely an option worth pursuing. However, this will inevitably increase the time required to complete a scan by roughly five to ten minutes. Protocol modification must be carefully designed and evaluated with clinical trials to ensure the scan efficiency and quantitative reliability.

The present study has several limitations. First, we used cardiac MRI as the reference of MBF quantification, rather than cardiac PET, which other investigators would have typically opted for [13, 41, 42]. We chose to use cardiac MRI over PET simply because cardiac MRI is easier to perform than cardiac PET in our institute due to technical reasons. Cardiac MRI reduces the radiation exposure of the test subjects. Moreover, wall motion and the presence of old infarctions can be evaluated using cardiac MRI. However, the uptake mechanism of PET perfusion tracers is more similar to that of ^99m^Tc-Sestamibi, as compared to Gd-DTPA. Gd-DTPA is an extracellular agent, which may explain the difference between SPECT- and MR-measured MBF. Furthermore, cardiac MRI typically acquires up to 4–5 slices over the whole heart, whereas PET acquires approximately 20–30 slices in a similar manner as SPECT. Thus, cardiac PET may be more suitable as a reference to validate the SPECT-measured MBF. Second, in our study, only 13% of the subjects (4/30) underwent invasive CAG. Hence, we could not use CAG-measured coronary stenosis as the reference, unlike many previous studies that correlated image-based flow quantification with the degree of coronary stenosis [8, 12, 14]. Third, since cardiac MR provides fewer short-axis slices than SPECT, a discrepancy in ROI placement is inevitable. Although we used the same ROI contours and attempted to place the ROIs consistently, ROI placement is inherently imperfect and some bias is inevitable when comparing measurements between two modalities. Finally, we could only use images reconstructed with the scanner’s built-in software, and hence, image correction is limited. For example, we were unable to perform scatter and attenuation correction. The effects of those image corrections on MBF quantification require further investigation.

## Conclusion

In the present study, we found that it is feasible to perform a rapid, dynamic and single-scan rest/stress SPECT MPI, with the capability of hyperemic MBF quantification. With a short scan time of 24 minutes, images of the rest and stress portions can both be obtained, along with hyperemic MBF measurements. Our data show that SPECT-measured hyperemic MBF is strongly correlated with the MR-measured MBF. Moreover, we found that SPECT-measured hyperemic MBF can reliably detect coronary ischemia, as compared to MR-measured CFR. Based on these findings, it is possible to obtain a high-quality measurement of the hyperemic MBF with our rapid, single-scan protocol designed for CZT-based SPECT cameras. Although further studies are required to establish a clinical standard, this protocol and the related data processing methods can serve as a template for developing routine SPECT MPI with flow quantification capabilities.

## Supporting information

S1 File Inclusion and exclusion criteria of the trial. Detailed description for the inclusion and exclusion criteria for subjects involved in this study.
(PDF)

S2 File Supplemental figures for data analysis and evaluation. S2 File contains additional graphs to illustrate the curve fitting details, the gender-specific statistical graphs, and the Bland-Altman plots of the CAG and non-CAG groups.
(PDF)

S3 File TREND statement checklist. (PDF)

S4 File Trial protocol. The trial protocol for this study in Chinese and English.
(PDF)

## Abbreviations

CZT: cadmium zinc telluride
CAD: coronary artery disease
CAG: coronary angiography
CFR: coronary flow reserve
DCE: dynamic contrast-enhanced
MBF: myocardial blood flow
MPI: myocardial perfusion imaging
PCI: percutaneous coronary intervention
SPECT: single-photon emission computed tomography
