# Supplementary material for: Single-scan rest/stress imaging with 99mTc-Sestamibi and cadmium zinc telluride-based SPECT for hyperemic flow quantification: A feasibility study evaluated with cardiac magnetic resonance imaging
Source: PLoS One. 2017 Aug 17;12(8):e0183402. doi: 10.1371/journal.pone.0183402 (PMC5560722; doi:10.1371/journal.pone.0183402)
Supplement: S1 File — Detailed description for the inclusion and exclusion criteria for subjects involved in this study. (PDF) [file pone.0183402.s001.pdf]

- Inclusion criteria

1. Subjects with high possibility of coronary artery disease after being evaluated by a cardiologist.
2. > 20 years old.
3. Not under infection or other conditions inappropriate for being recruited.
4. Without myocardial infarction within one week.
5. With stable blood pressure.
6. Subjects with normal kidney functions and without allergic reactions to MRI contrast agents.

- Exclusion criteria

1. Subjects who cannot take a SPECT scan or do not agree to sign the informed consent.
2. Pregnant subjects or subjects who are currently breast-feeding.
3. Those currently under radiation therapies.
4. Patients with pleural effusion.
5. Patients who are allergic to SPECT tracers.
6. Patients with serious psychological disorders.
7. Patients with asthma.
8. Patients with stroke within a week.
9. Patients with AV block.
10. Patients with sinus syndrome.
11. Subjects who are currently under other clinical trials.
12. Subjects with poor kidney functions who are inappropriate for MRI contrast agents.
13. Subjects with pacemakers.
14. Subjects with Claustrophobia.
15. Subjects who cannot hold breath for cardiac MRI studies.
