# Supplementary material for: Single-scan rest/stress imaging with 99mTc-Sestamibi and cadmium zinc telluride-based SPECT for hyperemic flow quantification: A feasibility study evaluated with cardiac magnetic resonance imaging
Source: PLoS One. 2017 Aug 17;12(8):e0183402. doi: 10.1371/journal.pone.0183402 (PMC5560722; doi:10.1371/journal.pone.0183402)
Supplement: S2 File — contains additional graphs to illustrate the curve fitting details, the gender-specific statistical graphs, and the Bland-Altman plots of the CAG and non-CAG groups. (PDF) [file pone.0183402.s002.pdf]

S2 File. Supplemental figures for the data analysis and evaluation

Detailed processing procedures of the time-intensity curves from MR and SPECT

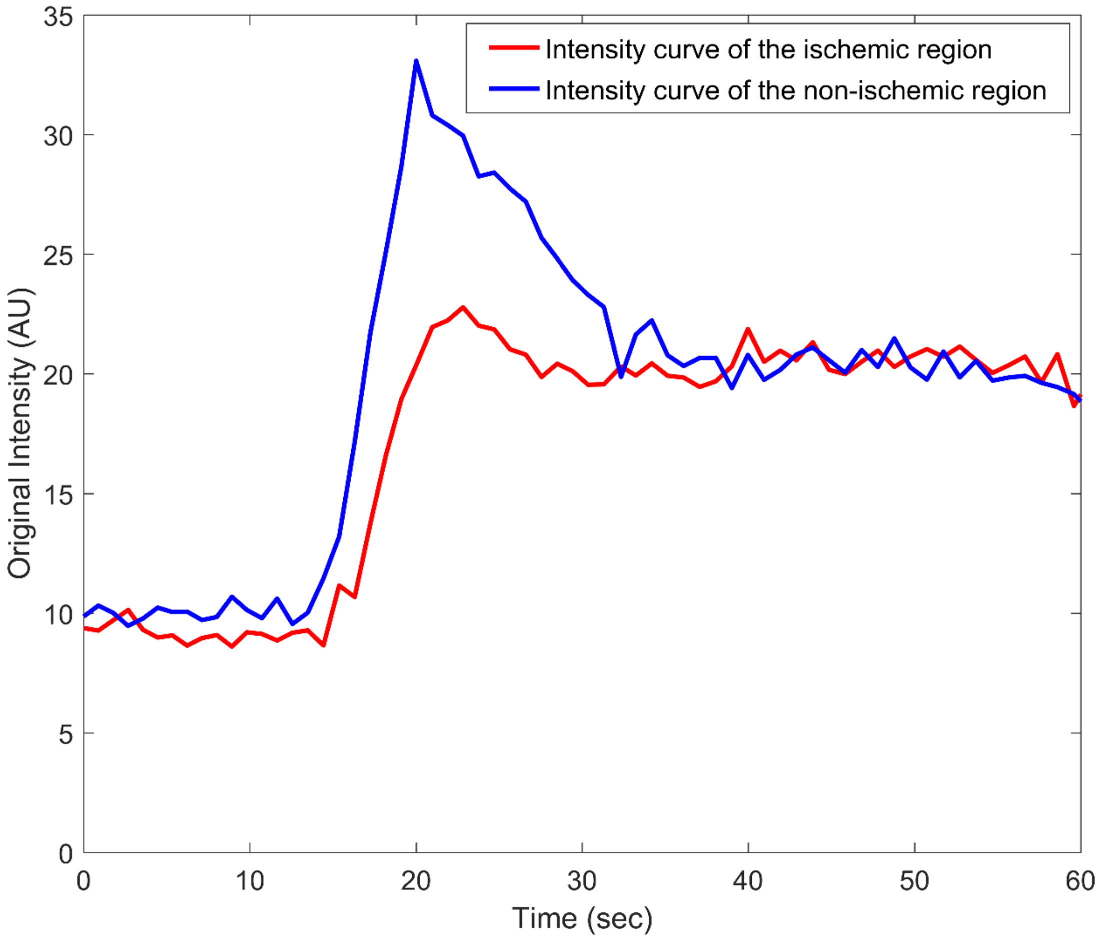

S2 Fig A. The raw MR time-intensity curves of the subject shown in Fig 3.

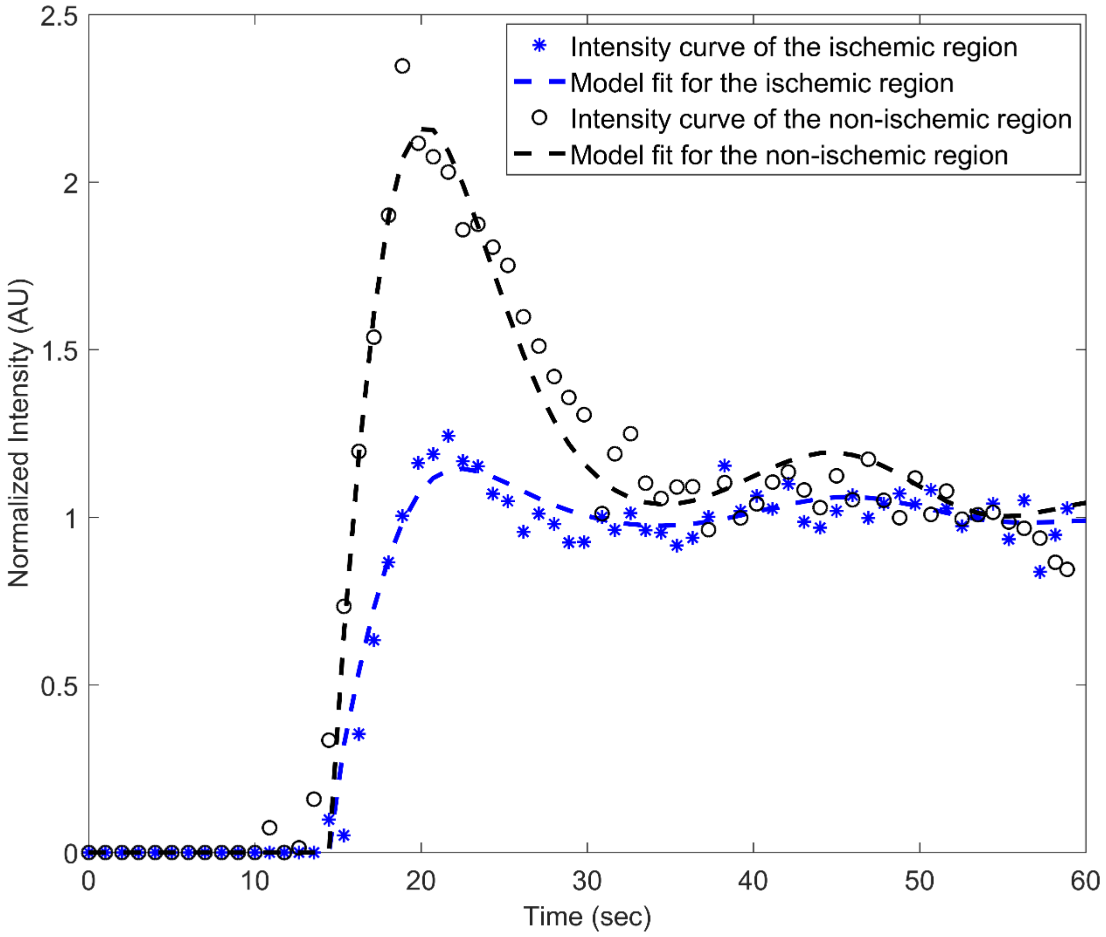

S2 Fig B. The normalized MR time-intensity curves and their model fits of the subject shown in Fig 3.

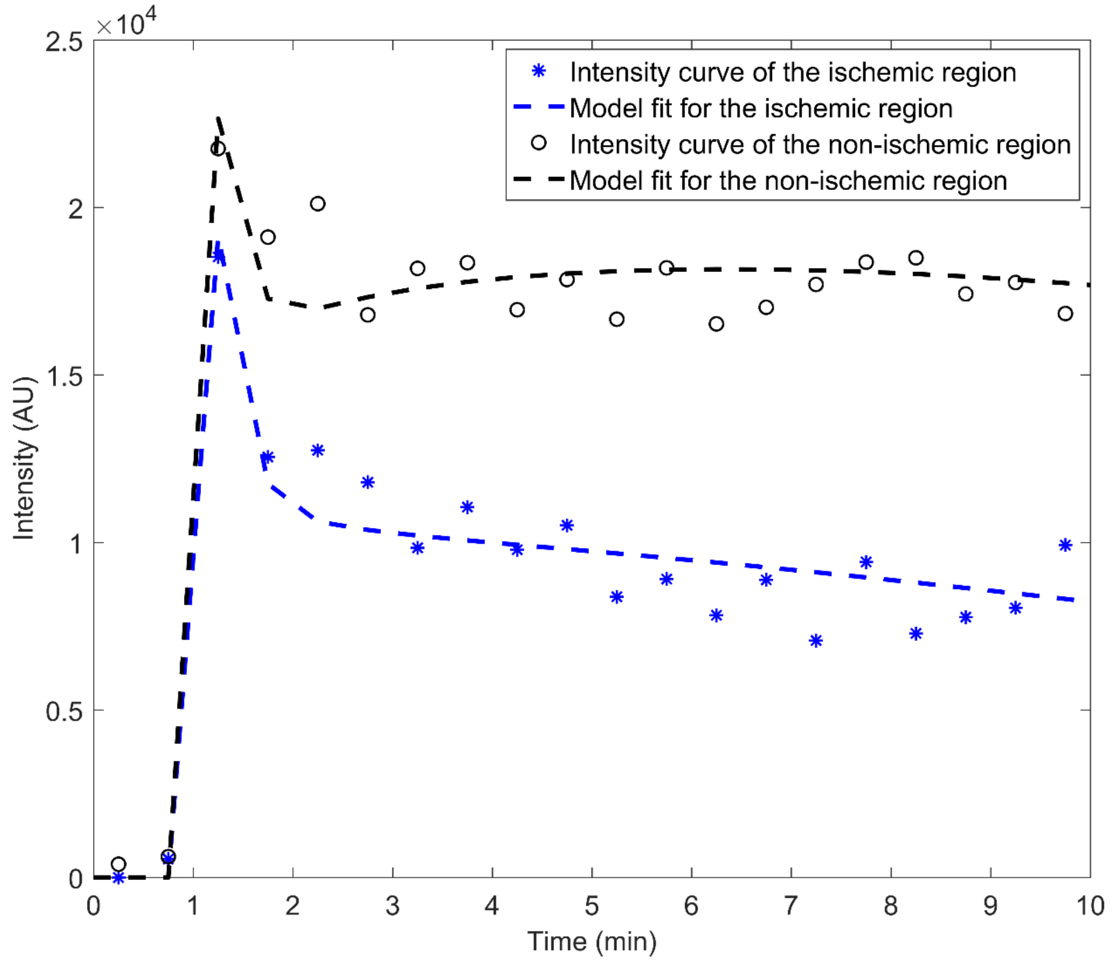

S2 Fig C. The SPECT time-activity curves and their model fits of the subject shown in Fig 3.

Gender-specific analytical plots of the estimated MBF and CFR

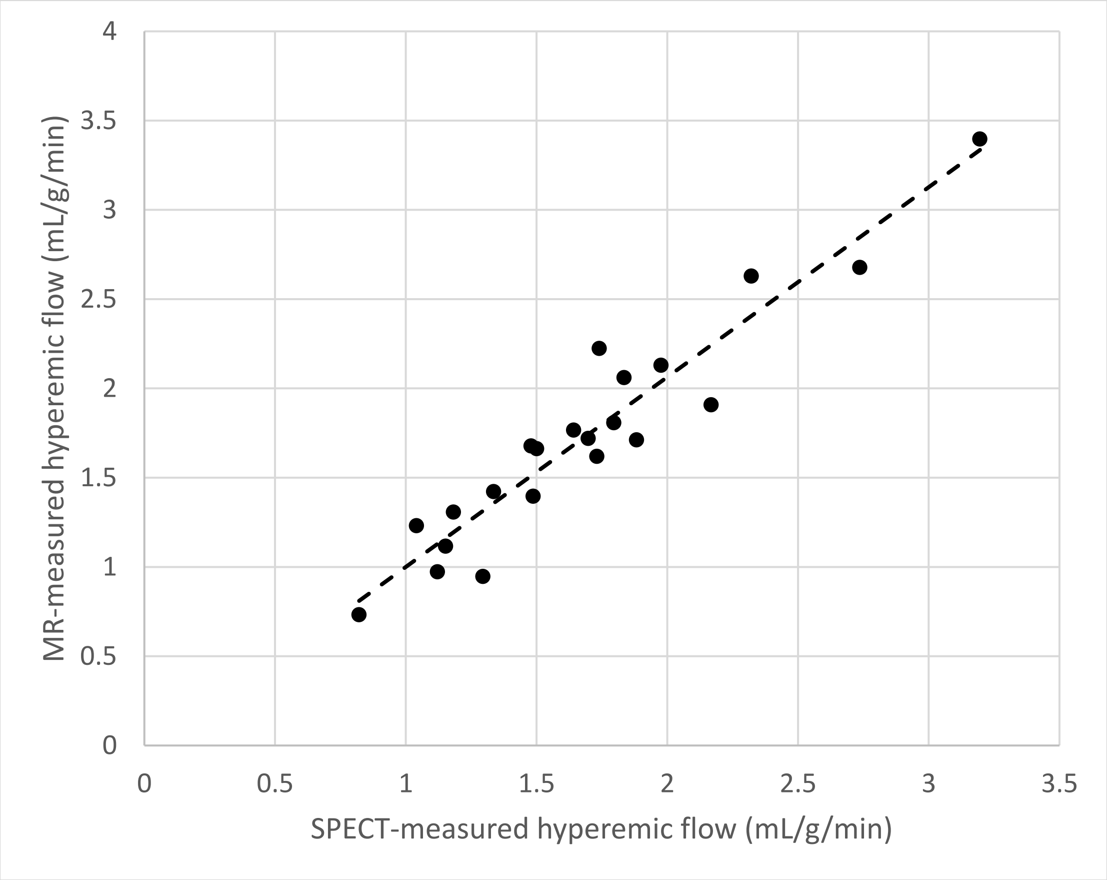

S2 Fig D. Scatter plot of the SPECT-measured and MR-measured MBF in female subjects. Pearson's correlation coefficient r^2=0.90. The slope was 1.06.

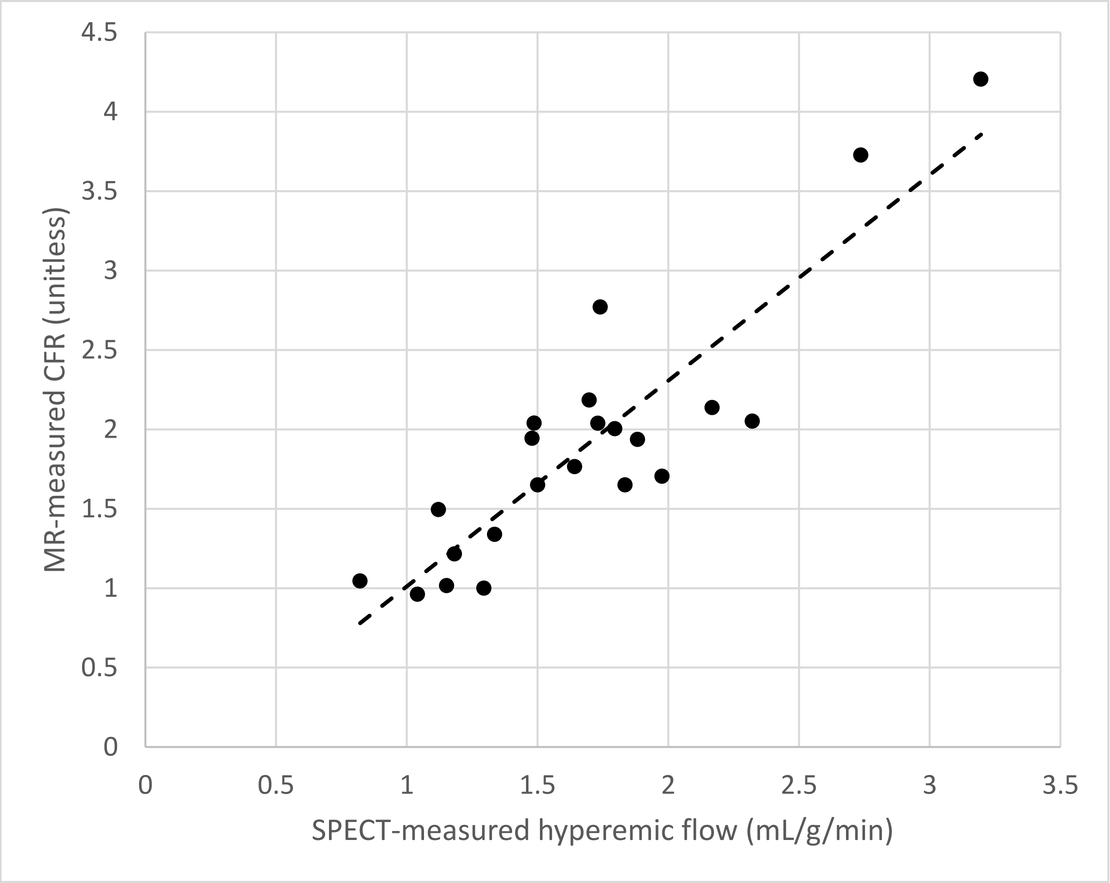

S2 Fig E. Scatter plot of the SPECT-measured and MR-measured MBF in male subjects. Pearson's correlation coefficient r^2=0.66. The slope was 0.87.

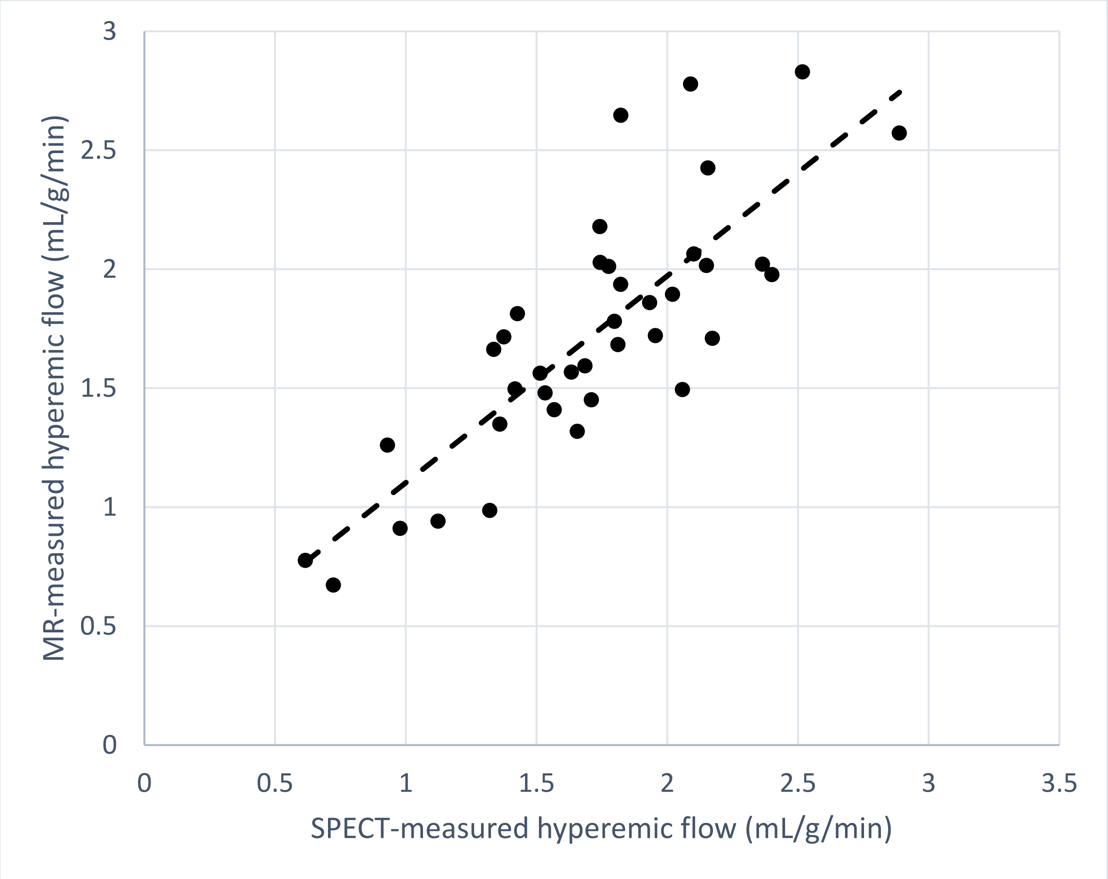

S2 Fig F. Scatter plot of the SPECT-measured MBF and MR-measured CFR in female subjects. Pearson's correlation coefficient r^2=0.79. The slope was 1.29.

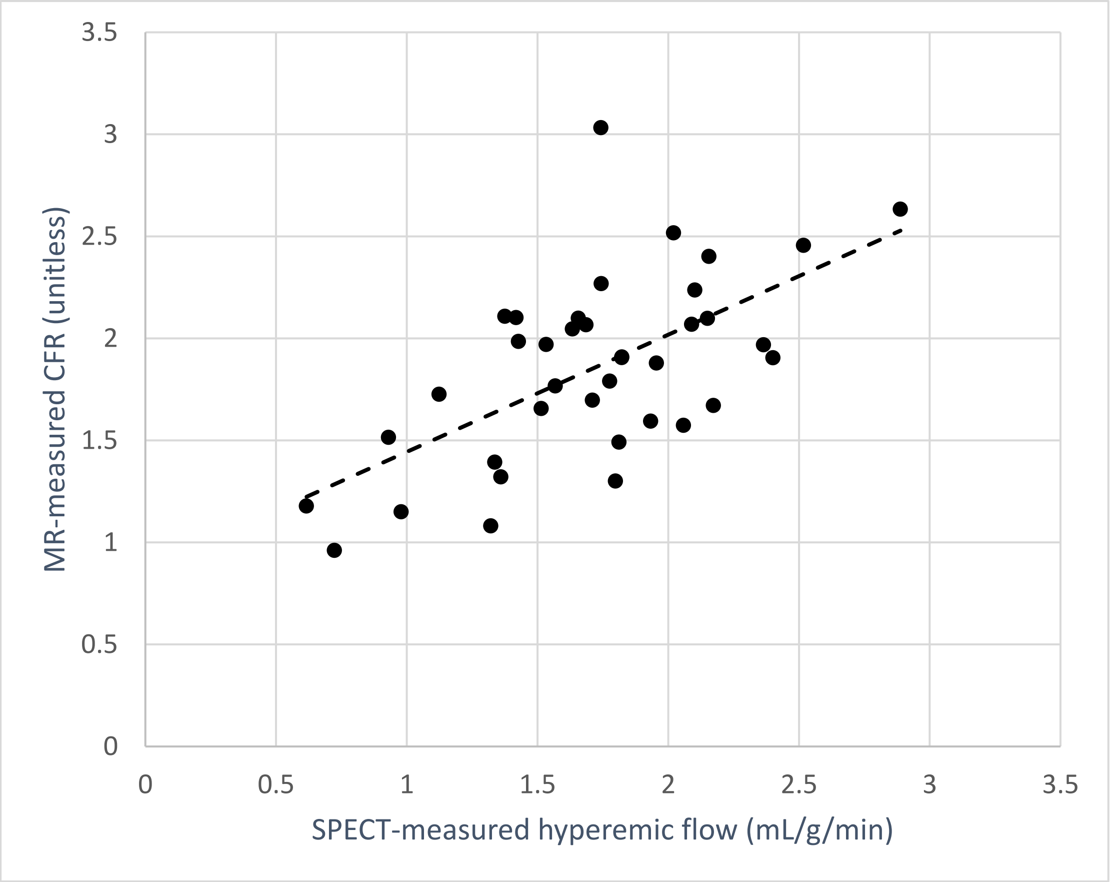

S2 Fig G. Scatter plot of the SPECT-measured MBF and MR-measured CFR in male subjects. Pearson's correlation coefficient r^2=0.38. The slope was 0.57.

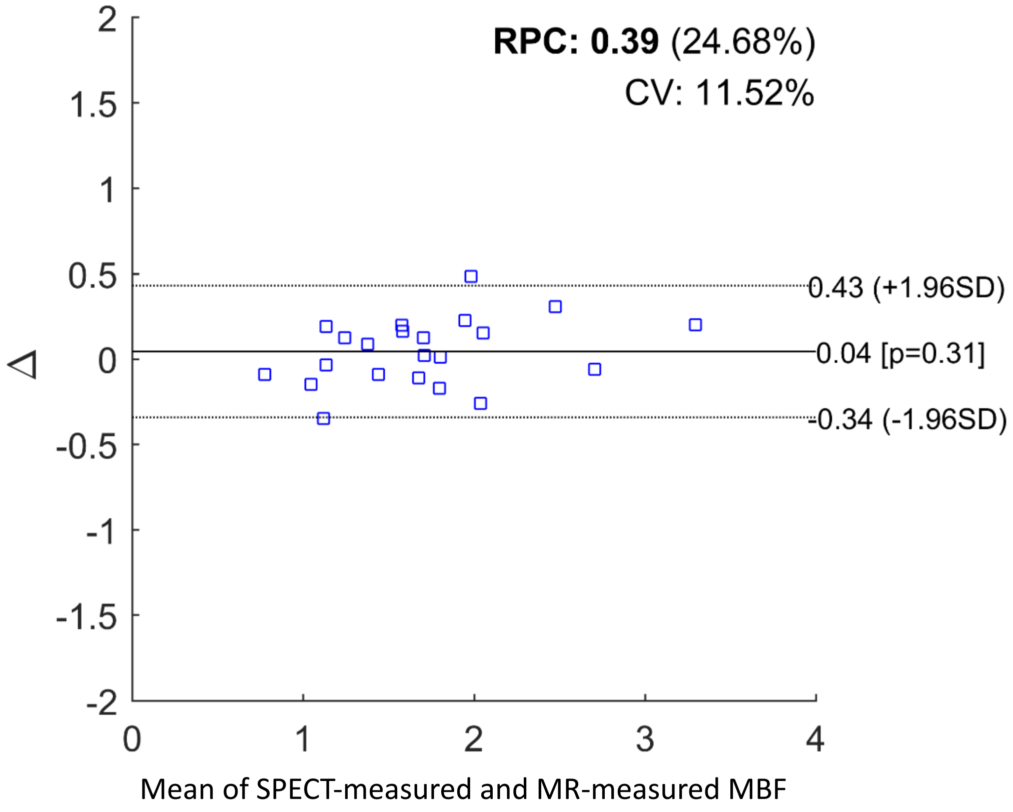

S2 Fig H. Bland-Altman plot of the SPECT-measured and MR-measured MBF in female subjects. △ represents the difference between those two parameters.

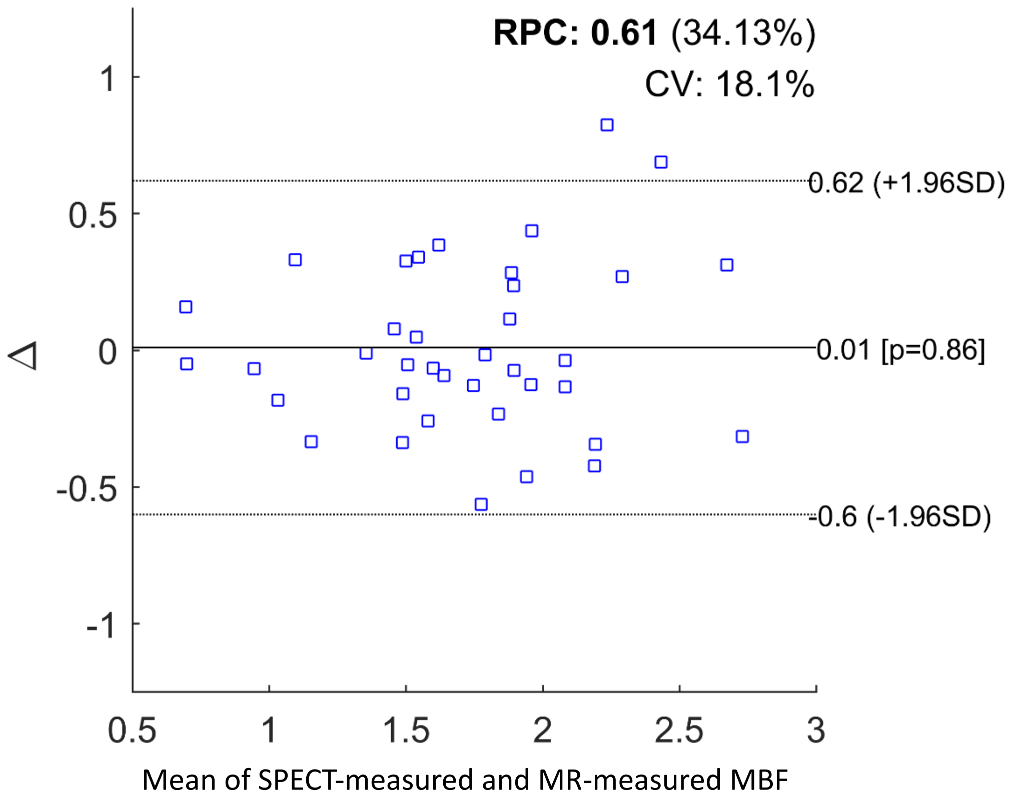

S2 Fig I. Bland-Altman of the SPECT-measured and MR-measured MBF in male subjects. △ represents the difference between those two parameters.

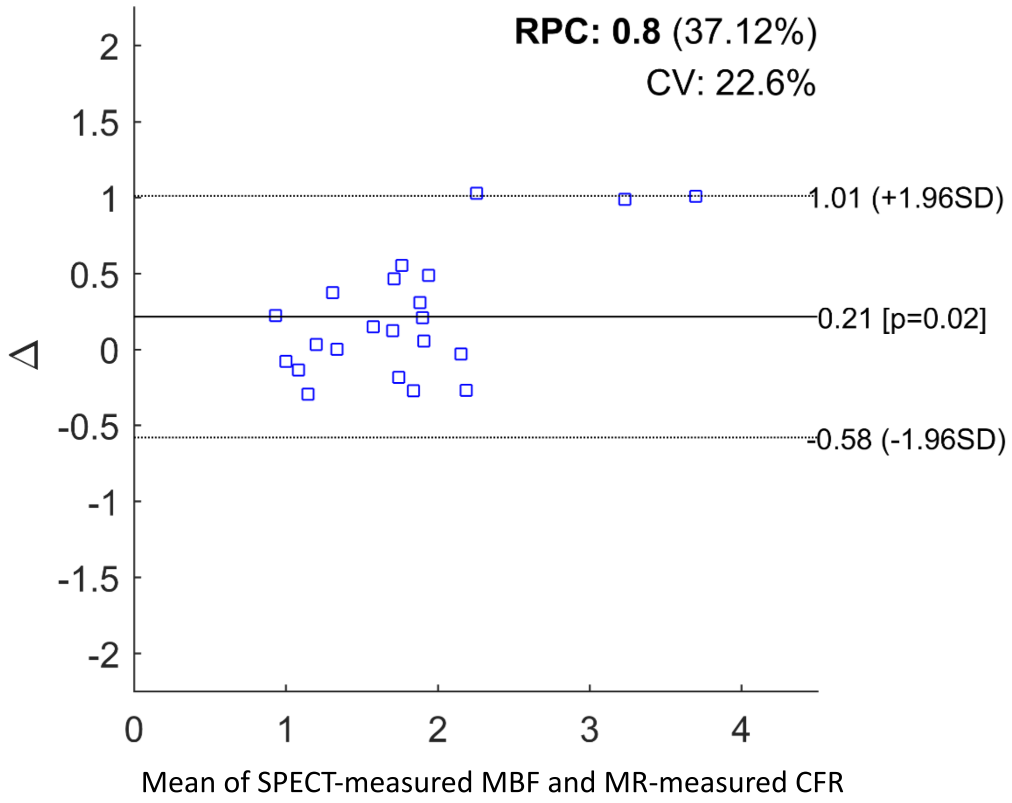

S2 Fig J. Bland-Altman of the SPECT-measured MBF and MR-measured CFR in female subjects. △ represents the difference between those two parameters.

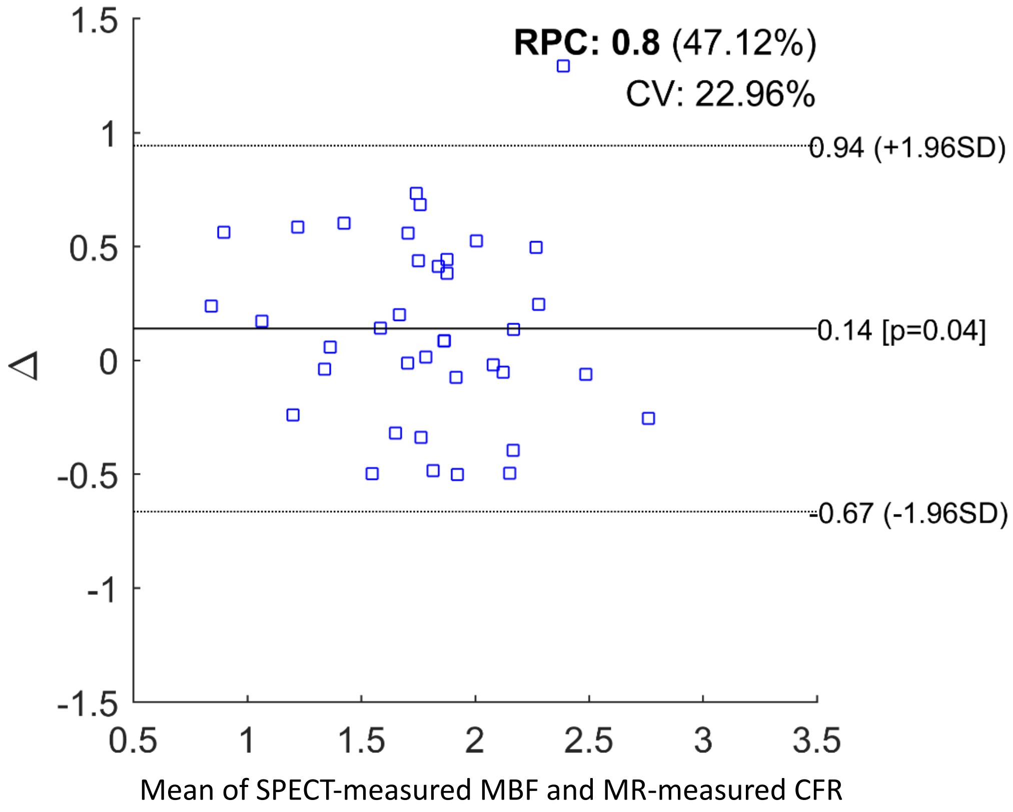

S2 Fig K. Bland-Altman of the SPECT-measured MBF and MR-measured CFR in male subjects. △ represents the difference between those two parameters.

Bland-Altman plots of the estimated MBF and CFR in the groups of subjects who did and did not receive the CAG exams

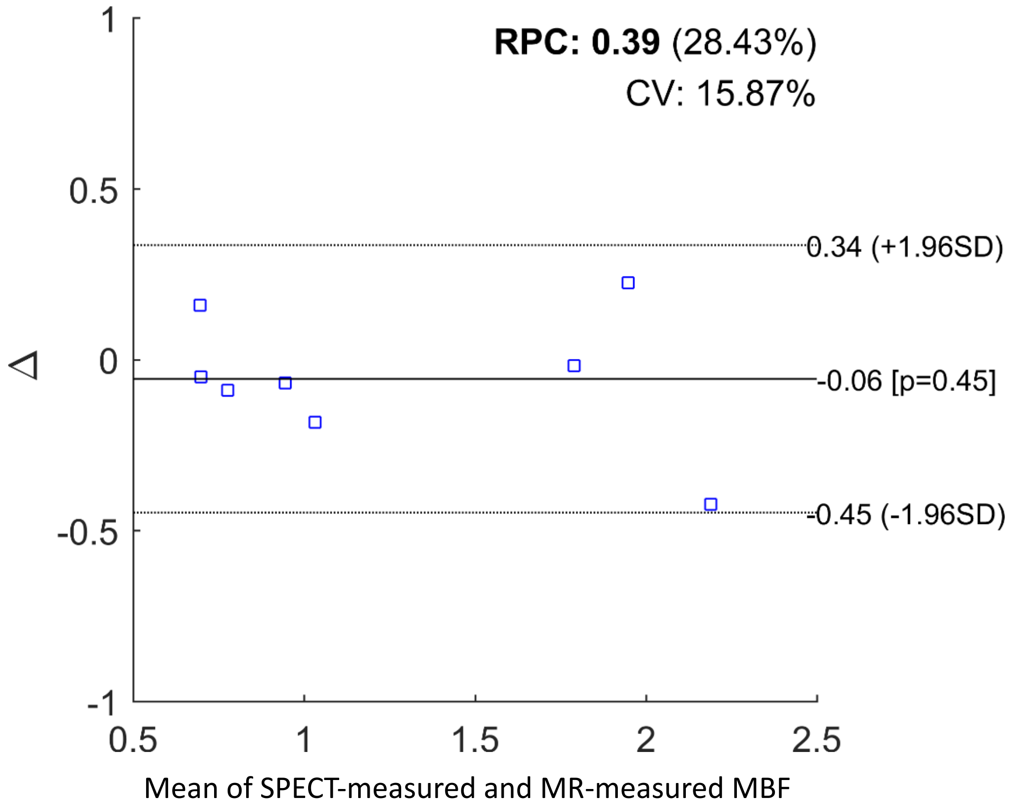

S2 Fig L. Bland-Altman of the SPECT-measured and MR-measured MBF in subjects who underwent CAG exams. △ represents the difference between those two parameters.

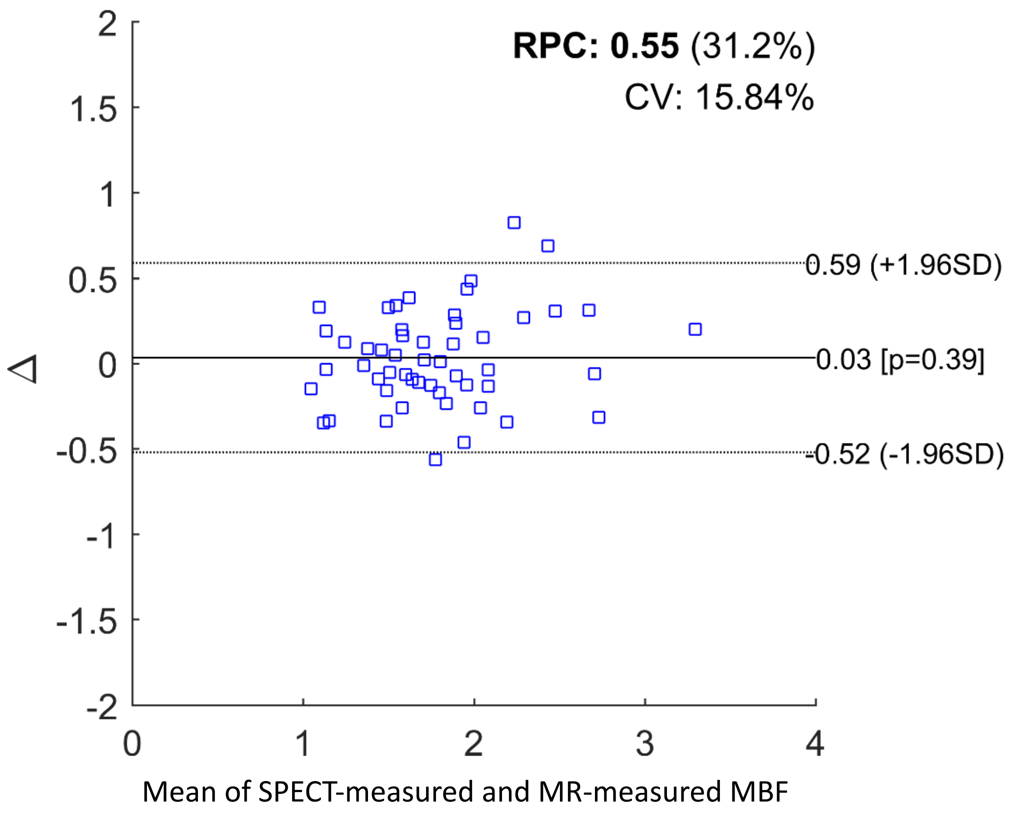

S2 Fig M. Bland-Altman of the SPECT-measured and MR-measured MBF in subjects who did not underwent CAG exams. △ represents the difference between those two parameters.

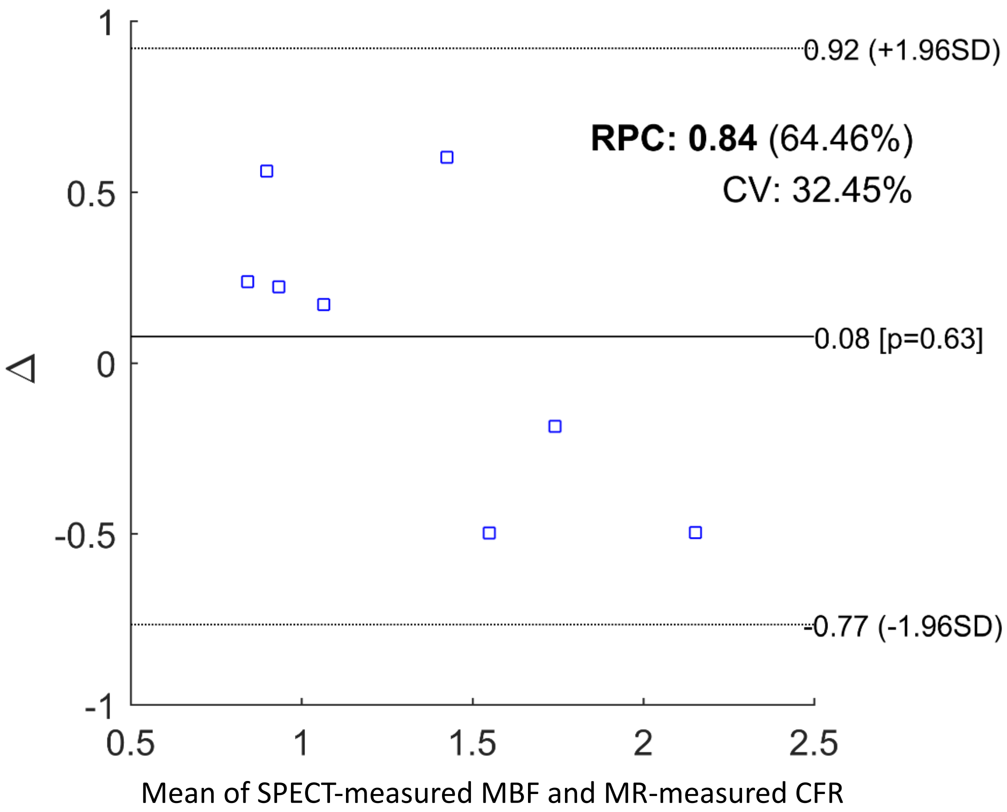

S2 Fig N. Bland-Altman of the SPECT-measured MBF and MR-measured CFR in subjects who underwent CAG exams. △ represents the difference between those two parameters.

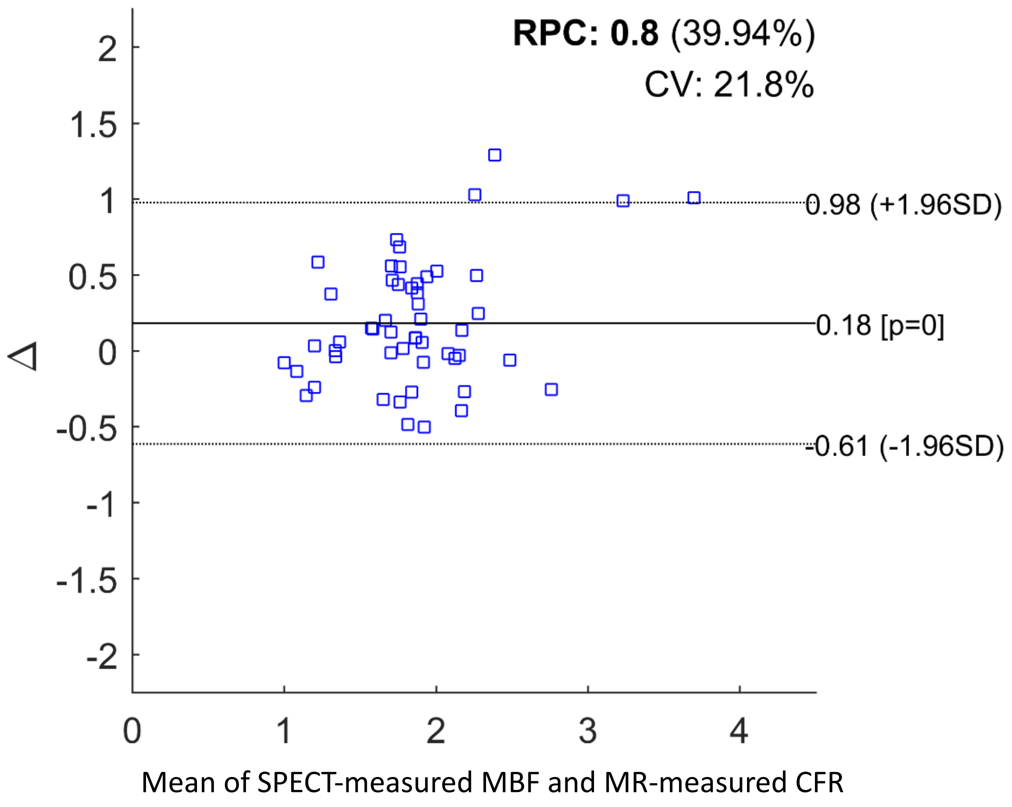

S2 Fig O. Bland-Altman of the SPECT-measured MBF and MR-measured CFR in subjects who did not underwent CAG exams. △ represents the difference between those two parameters.
