# Supplementary material for: Single-scan rest/stress imaging with 99mTc-Sestamibi and cadmium zinc telluride-based SPECT for hyperemic flow quantification: A feasibility study evaluated with cardiac magnetic resonance imaging
Source: PLoS One. 2017 Aug 17;12(8):e0183402. doi: 10.1371/journal.pone.0183402 (PMC5560722; doi:10.1371/journal.pone.0183402)
Supplement: S4 File — The trial protocol for this study in Chinese and English. (PDF) [file pone.0183402.s004.pdf]

試驗計畫書

Trial Study Protocol

Single-scan rest/stress imaging with cadmium zinc telluride-based SPECT for coronary flow quantification

I. 主題

使用新的影像分析技術，改進單光子斷層心臟造影流程，對冠狀動脈血流進行定量分析

Target:

Improving the SPECT imaging procedures with novel image analysis techniques for quantitative analysis of coronary blood flow.

II. 研究背景（含國際研究現況）、研究目的。

Research background

研究背景 (Background)

近年來，心血管疾病已成為目前全球及台灣最大的健康威脅。心血管疾病根據 WHO (World Health Organization)的統計，是每年人類死因的第一位，以 2008 年為例，因心血管疾病死亡的人數比例就佔了近百分之三十，這個現象在未開發和開發中國家尤其明顯，推測與醫療的可近性 (Healthcare Access) 和品質有關係。然而，儘管台灣目前是已開發國家，在中央健康保險局 2009 年的統計當中，心血管疾患約佔了門診人數的 20%，住院人數的 31.63%；在 2010 年心血管疾病甚至高居十大死因的第二位，心血管疾病的重要性可見一班。

Cardiovascular diseases have become the top threat to the human health. According the WHO, cardiovascular diseases claim the top cause of death globally for 30% of mortality. In Taiwan, cardiovascular is the second highest cause of death based on the statistics of Department of Health in 2009. Although Taiwan is a developed country, 20% outpatient and more than 30% inpatient cost is spent on the healthcare of cardiovascular disease patients. The fraction of cardiovascular disease patients is still growing each year and therefore remains a critical public health issue in Taiwan and the world.

心血管疾病當中，又以冠狀動脈疾病最容易早期發現、早期治療，許多報告指出，越早期治療，越能有效幫助心肌功能的恢復，所以冠狀動脈疾病相對於其他疾病來說，有較複雜也較嚴格的時機點和檢查／治療標準，其中最重要的指引源自於美國心臟科學會 (American Heart Association，AHA)。過去發展檢查／治療最重要的里程碑之一就是心導管 (cardiac catheterization)，AHA 整理了美國 1979 年至 2005 年心導管施作的情形，其數量成長了 342%，但是心導管為一侵入性的檢查／治療工具，風險性相對較高，AHA 統計了美國 2005 年接受心導管患者發生重大併發症，包括對顯影劑有過敏性休克、心因性休克、急性冠心症、急性心臟衰竭、心包膜填塞、腎臟衰竭，其機率平均為 1.3% (lowest quartile: 0.2，highest quartile: 1.8%)。所以目前，診斷冠狀動

脈疾病的工具以非侵入性的檢查為主，而非心導管。

Among different cardiovascular diseases, coronary artery diseases (CAD) are the most frequently and one of the most deadly diseases. In a recent report made by the American Heart Association (AHA) [1], CAD possesses 50-70% of all heart disease cases (Figure 2). Fortunately, CAD has a good chance of being treated if diagnosed as early as possible. The most standard treatment of CAD is through cardiac catheterization for balloon angioplasty and stent placement. Although catheterization can also be used for diagnostic purposes, due to its invasive nature it is inappropriate for first-line diagnosis. In fact, AHA has reported a high complication rate of 1.3% for catheterization, with potential side effects including *anaphylactic* reactions to the contrast medium, acute ischemia, acute heart failure and kidney failure [1]. Therefore, current mainstay of CAD diagnostic tools mainly encompass non-invasive examinations. Once the patient is determined with a high risk of CAD through non-invasive approaches, catheterization will then be used to first confirm the stenosis and then further used to perform the angioplasty or stent treatments.

單光子斷層影像(SPECT)目前仍可說是心臟影像檢查的主要選項之一。SPECT 相較於正子斷層影像(PET)，SPECT 儀器與藥物較為便宜，但一個限制在於無法進行定量分析，在心臟檢查中，無法以定量的方式量測心臟血液灌流量。

Among different cardiac imaging modalities, SPECT remains to be the main stream. Compared to PET, SPECT is much cheaper in the instruments and tracers. However the limitation of SPECT is the lack of quantification capabilities.

研究目的 (Research aims)

為改善 SPECT 心臟檢查的定量分析能力，我們將提出一個經改良過的 SPECT 造影流程，並發展使用動態模組分析為基礎的影像處理方式，量測冠狀動脈血流。本臨床試驗的主要目的，是驗證新發展的 SPECT 影像分析技術，其量測的 CFR 是否與 MRI 所量測的 CFR 具有相同的正確性。

In order to improve the quantitative capabilities, we propose a modified SPECT protocol with its processing methods based on kinetic modeling to measure the coronary blood flow. The purpose of this trial is to evaluate whether the measured flow is accurate compared to the measurement with cardiac MRI.

III. 方法(Methods)：

(1) 人體研究者（以下稱受試者）之條件（納入、排除條件）、招募方法及數目。

Human subjects (called subjects in the following) inclusion and exclusion criteria:

(A) 受試者相關資料 (Subject information)：

研究中，我們計畫納入 100 位冠狀動脈疾病患來作研究。

病患納入標準為：

1. 經心臟科醫師診斷，高度懷疑阻塞性冠狀動脈疾病，並經運動心電圖評估，心臟科醫師認為需進行心臟影像檢查，以評估是否進行心導管治療者。
2. 年齡大於 20 歲。
3. 不在感染狀態，並無其他的病症而不適合進入研究，例如，未控制之高血壓，鬱積性心臟衰竭。
4. 最近一周無罹患心肌梗塞者。
5. 無血壓不穩定者如低血壓（收縮壓小於 80mmHg）、嚴重高血壓（收縮壓大於 180mmHg）等，
6. 對於磁振造影對比劑無過敏反應，且無不正常腎臟功能者。

In this study we plan to recruit 100 subjects with suspected CAD. The inclusion criteria is:

1. Subjects with high possibility of coronary artery disease after being evaluated by a cardiologist.
2. > 20 years old.
3. Not under infection or other conditions inappropriate for being recruited.
4. Without myocardial infarction within one week.
5. With stable blood pressure.
6. Subjects with normal kidney functions and without allergic reactions to MRI contrast agents.

排除標準為：

1. 無法配合進行單光子斷層掃描與未能簽署同意書的病患。
2. 懷孕或是哺乳的病患。
3. 正在進行放射線治療。
4. 主要以惡性胸水表現而無其他可測量性病灶的病患。
5. 對於輻射追蹤劑有過敏者。
6. 具有嚴重神經或是精神疾病病患，例如，老年痴呆因而不能了解且簽署同意書之病患。
7. 有急性氣喘或是局限性／阻塞性肺病急性發作者。
8. 最近一周有腦中風之病史。
9. 有二度以上心房心室傳導阻滯者。
10. 有病竇症候群，並且尚未安裝心律調節器者。
11. 正在進行其他試驗性藥物治療者。
12. 腎功能不佳，不適合使用磁振造影對比劑者。
13. 安裝有心臟節律器及其他金屬植入物，經評估不適合進行磁振造影者。
14. 有密室恐懼症而無法接受磁振造影檢查者。
15. 無法配合閉氣做磁振造影者。

Exclusion criteria:

1. Subjects who cannot take a SPECT scan or do not agree to sign the informed consent.
2. Pregnant subjects or subjects who are currently breast-feeding.
3. Those currently under radiation therapies.
4. Patients with pleural effusion.
5. Patients who are allergic to SPECT tracers.
6. Patients with serious psychological disorders.
7. Patients with asthma.
8. Patients with stroke within a week.
9. Patients with AV block.
10. Patients with sinus syndrome.
11. Subjects who are currently under other clinical trials.
12. Subjects with poor kidney functions who are inappropriate for MRI contrast agents.
13. Subjects with pacemakers.
14. Subjects with Claustrophobia.
15. Subjects who cannot hold breath for cardiac MRI studies.

(B) 如何招募或接觸到受試者（含實驗組及對照組）(Subjects recruitment)
由心臟科醫師診斷後，評估適合參與實驗、高度懷疑阻塞性冠狀動脈疾病且安排住院做心導管病人，詢問意願並招募。

The cardiologist will refer subjects who are suspected with CAD and appropriate for this trial.

(C) 樣本數 (Expected sample)
100

(2) 試驗設計及方法 Trial design and methods

在進行心導管前，先進行一次 rest/stress ^{99m}Tc-MIBI SPECT 檢查，再進行一次 rest/stress cardiac MRI 檢查。病人影像檢查經評估後，決定是否進行心導管檢查；如進行心導管檢查，則由心導管檢查結果，決定。於心導管程序完成後，後續的追蹤依目前臨床標準程序進行。在 SPECT 方面，每次 rest-stress ^{99m}Tc-MIBI SPECT 的檢查程序示意圖如下：

All subjects will receive a rest/stress 99mTc-MIBI SPECT study and then a rest/stress cardiac MRI. After the evaluation by cardiologists, decision of whether coronary angiography is need will be made by the cardiologist. After the coronary angiography, standard medical follow-up will be conducted. For each SPECT scan, the protocol follows:

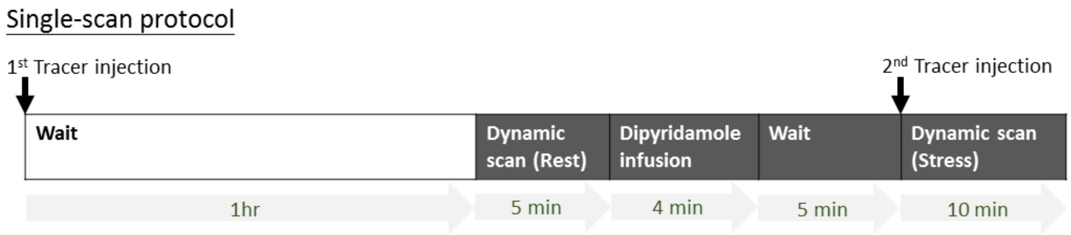

每次 rest-stress cardiac MRI 的檢查程序示意圖如下：

The cardiac MRI follows:

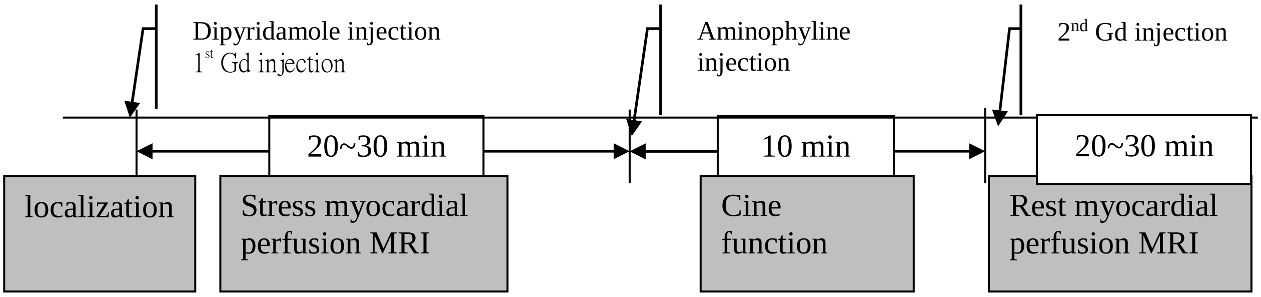

使用劑量 Dosage of drugs/tracers

Dipyridamole	0.56 mg/kg	4-min slow iv. infusion
1st injection ^{99m}Tc-MIBI	10 mCi	Bolus, i.v.
2nd injection ^{99m}Tc-MIBI	20 mCi	Bolus, i.v.
1st injection Magnevist	0.1mmol/kg	Bolus, i.v.
2nd injection Magnevist	0.1mmol/kg	Bolus, i.v.

試驗設計參考文獻 (Reference on protocol design)：

1. El Fakhri G, Kardan A, Sitek A, Dorbala S, Abi-Hatem N, Lahoud Y, Fischman A, Coughlan M, Yasuda T, Di Carli MF. Reproducibility and accuracy of quantitative myocardial blood flow assessment with (82)rb pet: Comparison with (13)n-ammonia pet. *Journal of nuclear medicine : official publication, Society of Nuclear Medicine*. 2009;50:1062-1071
2. Khorsand A, Graf S, Pirich C, Muzik O, Kletter K, Dudczak R, Maurer G, Sochor H, Schuster E, Porenta G. Assessment of myocardial perfusion by dynamic n-13 ammonia pet imaging: Comparison of 2 tracer kinetic models. *Journal of nuclear cardiology : official publication of the American Society of Nuclear Cardiology*. 2005;12:410-417
3. Krivokapich J, Smith GT, Huang SC, Hoffman EJ, Ratib O, Phelps ME, Schelbert HR. 13n ammonia myocardial imaging at rest and with exercise in normal volunteers. Quantification of absolute myocardial perfusion with dynamic positron emission

tomography. *Circulation*. 1989;80:1328-1337
4. Taillefer R, Gagnon A, Laflamme L, Grégoire J, Léveillé J, Phaneuf DC. Same day injections of tc-99m methoxy isobutyl isonitrile (hexamibi) for myocardial tomographic imaging: Comparison between rest-stress and stress-rest injection sequences. *European Journal of Nuclear Medicine and Molecular Imaging*. 1989;15:113-117

(3) 執行期間及預計進度 (Expected timeline and progress)。

2012 年 04 月 01 日至 2015 年 3 月 31 日止。預計每個月約收集三名受試者資料。

4/1/2012~3/31/2015. Expected three subjecs/month.

(4) 研究結果之評估方式(Evaluation)

我們將對新發展的影像分析方法評估定量分析效果。

We will evaluate the analytical performances of the newly developed analysis methods.

(5) 預期效果、統計方法。(Expected effect and statistical methods)

我們預期使用新發展的影像分析方法，可以達到與 cardiac MRI 影像所得到的定量分析相似的正確性，並提高生理參數影像的影像品質。我們會計算以 cardiac MRI 和新式 SPECT 影像分析方法，在心臟不同的 coronary territory 中，所計算的 blood flow，兩者間是否具有高度相關性。

We expect that the SPECT-measured flow will be similar to the MR-measured flow. Therefore we will evaluate the flow measure in different coronary territories and check the correlation between SPECT- and MR-measure values.

(6) 受試者之追蹤及必要之復健計畫。(Subject follow-up)

受試者將由心臟科醫師按一般臨床心導管手術程序，定期追蹤術後恢復情形。

The subjects will be followed up in clinical cardiology by the cardiologists according to the standard clinical procedures.

IV. 有關之國內、外已發表之文獻報告，或是文獻清單 (reference list)。

1. Rosamond W, Flegal K, Furie K, Go A, Greenlund K, Haase N, Hailpern SM, Ho M, Howard V, Kissela B, Kittner S, Lloyd-Jones D, McDermott M, Meigs J, Moy C, Nichol G, O'Donnell C, Roger V, Sorlie P, Steinberger J, Thom T, Wilson M and Hong Y. Heart disease and stroke statistics--2008 update: a report from the American Heart Association Statistics Committee and Stroke Statistics Subcommittee. *Circulation*. 2008;117:e25-146.
2. El Fakhri G, Kardan A, Sitek A, Dorbala S, Abi-Hatem N, Lahoud Y, Fischman A, Coughlan M, Yasuda T and Di Carli MF. Reproducibility and accuracy of quantitative myocardial blood flow assessment with (82)Rb PET: comparison with (13)N-ammonia PET. *Journal of nuclear medicine : official publication, Society of Nuclear Medicine*. 2009;50:1062-71.

3. Khorsand A, Graf S, Pirich C, Muzik O, Kletter K, Dudczak R, Maurer G, Sochor H, Schuster E and Porenta G. Assessment of myocardial perfusion by dynamic N-13 ammonia PET imaging: comparison of 2 tracer kinetic models. *Journal of nuclear cardiology : official publication of the American Society of Nuclear Cardiology*. 2005;12:410-7.
4. Krivokapich J, Smith GT, Huang SC, Hoffman EJ, Ratib O, Phelps ME and Schelbert HR. 13N ammonia myocardial imaging at rest and with exercise in normal volunteers. Quantification of absolute myocardial perfusion with dynamic positron emission tomography. *Circulation*. 1989;80:1328-37.
5. Ayalew A, Marie PY, Menu P, Mertes PM, Audonnet S, Jouan V, Olivier P, Karcher G, Ungureanu-Longrois D and Bertrand A. 201Tl and 99mTc-MIBI retention in an isolated heart model of low-flow ischemia and stunning: evidence of negligible impact of myocyte metabolism on tracer kinetics. *Journal of Nuclear Medicine*. 2002;43:566.
6. Bateman TM, Heller GV, McGhie AI, Friedman JD, Case JA, Bryngelson JR, Hertenstein GK, Moutray KL, Reid K and Cullom SJ. Diagnostic accuracy of rest/stress ECG-gated Rb-82 myocardial perfusion PET: comparison with ECG-gated Tc-99m sestamibi SPECT. *Journal of nuclear cardiology*. 2006;13:24-33.
7. Beanlands RSB, Muzik O, Melon P, Sutor R and Sawada S. Noninvasive quantification of regional myocardial flow reserve in patients with coronary atherosclerosis using nitrogen-13 ammonia positron emission tomography:: Determination of extent of altered vascular reactivity. *Journal of the American College of Cardiology*. 1995;26:1465-1475.
8. Bengel FM, Higuchi T, Javadi MS and Lautamaki R. Cardiac positron emission tomography. *Journal of the American College of Cardiology*. 2009;54:1-15.
9. Bocher M, Blevis IM, Tsukerman L, Shrem Y, Kovalski G and Volokh L. A fast cardiac gamma camera with dynamic SPECT capabilities: design, system validation and future potential. *Eur J Nucl Med Mol Imaging*. 2010;37:1887-902.
10. Cerqueira MD, Allman KC, Ficaro EP, Hansen CL, Nichols KJ, Thompson RC, Van Decker WA and Yakovlevitch M. Recommendations for reducing radiation exposure in myocardial perfusion imaging. *Journal of nuclear cardiology*. 2010;17:709-718.
11. Cerqueira MD, Weissman NJ, Dilsizian V, Jacobs AK, Kaul S, Laskey WK, Pennell DJ, Rumberger JA, Ryan T and Verani MS. Standardized myocardial segmentation and nomenclature for tomographic imaging of the heart. A statement for healthcare professionals from the Cardiac Imaging Committee of the Council on Clinical Cardiology of the American Heart Association. *The international journal of cardiovascular imaging*. 2002;18:539-42.
12. Chiao PC, Ficaro EP, Dayanikli F, Rogers WL and Schwaiger M. Compartmental analysis of technetium-99m-teboroxime kinetics employing fast dynamic SPECT at rest and stress. *Journal of nuclear medicine: official publication, Society of Nuclear Medicine*. 1994;35:1265.
13. Chow BJW, Ananthasubramaniam K, dekemp RA, Dalipaj MM, Beanlands RSB and Ruddy TD. Comparison of treadmill exercise versus dipyridamole stress with myocardial perfusion imaging using rubidium-82 positron emission tomography. *Journal of the American College of Cardiology*. 2005;45:1227-1234.
14. Conti A, Sammicheli L, Gallini C, Costanzo EN, Antoniucci D and Barletta G. Assessment of patients with low-risk chest pain in the emergency department: head-to-head comparison of exercise stress echocardiography and exercise myocardial SPECT. *American Heart Journal*. 2005;149:894-901.
15. El Fakhri G, Kardan A, Sitek A, Dorbala S, Abi-Hatem N, Lahoud Y, Fischman A, Coughlan M, Yasuda T and Di Carli MF. Reproducibility and accuracy of quantitative myocardial blood flow assessment with 82Rb PET: Comparison with 13N-ammonia PET.

Journal of Nuclear Medicine. 2009;50:1062.
16. El Fakhri G, Sitek A, Guérin B, Kijewski MF, Di Carli MF and Moore SC. Quantitative dynamic cardiac 82Rb PET using generalized factor and compartment analyses. *Journal of Nuclear Medicine*. 2005;46:1264.
17. El Fakhri G, Sitek A, Zimmerman RE and Ouyang J. Generalized five-dimensional dynamic and spectral factor analysis. *Medical physics*. 2006;33:1016.
18. Elhendy A, Bax JJ and Poldermans D. Dobutamine stress myocardial perfusion imaging in coronary artery disease. *Journal of nuclear medicine : official publication, Society of Nuclear Medicine*. 2002;43:1634-46.
19. Elhendy A, Schinkel A, Bax JJ, van Domburg RT and Poldermans D. Long-term prognosis after a normal exercise stress Tc-99m sestamibi SPECT study. *Journal of nuclear cardiology*. 2003;10:261-266.
20. Feng D, Huang SC and Wang X. Models for computer simulation studies of input functions for tracer kinetic modeling with positron emission tomography. *International journal of bio-medical computing*. 1993;32:95-110.
21. Flamen P, Bossuyt A and Franken PR. Technetium-99m-tetrofosmin in dipyridamole-stress myocardial SPECT imaging: intraindividual comparison with technetium-99m-sestamibi. *Journal of nuclear medicine: official publication, Society of Nuclear Medicine*. 1995;36:2009.
22. Friston KJ and Ashburner J. Statistical parametric mapping. *Functional neuroimaging: Technical foundations*. 1994:79-93.
23. Gupta NC, Esterbrooks DJ, Hilleman DE and Mohiuddin SM. Comparison of adenosine and exercise thallium-201 single-photon emission computed tomography (SPECT) myocardial perfusion imaging. *Journal of the American College of Cardiology*. 1992;19:248-257.
24. Hachamovitch R, Berman DS, Kiat H, Cohen I, Cabico JA, Friedman J and Diamond GA. Exercise myocardial perfusion SPECT in patients without known coronary artery disease: incremental prognostic value and use in risk stratification. *Circulation*. 1996;93:905-914.
25. Hachamovitch R, Berman DS, Kiat H, Cohen I, Lewin H and Amanullah A. Incremental prognostic value of adenosine stress myocardial perfusion single-photon emission computed tomography and impact on subsequent management in patients with or suspected of having myocardial ischemia. *The American Journal of Cardiology*. 1997;80:426-433.
26. Heller GV, Links J, Bateman TM, Ziffer JA, Ficaro E, Cohen MC and Hendel RC. American Society of Nuclear Cardiology and Society of Nuclear Medicine joint position statement: attenuation correction of myocardial perfusion SPECT scintigraphy. *Journal of nuclear cardiology*. 2004;11:229-230.
27. Jansen FP and Vanderheyden JL. The future of SPECT in a time of PET. *Nuclear medicine and biology*. 2007;34:733-735.
28. Khorsand A, Graf S, Pirich C, Muzik O, Kletter K, Dudczak R, Maurer G, Sochor H, Schuster E and Porenta G. Assessment of myocardial perfusion by dynamic N-13 ammonia PET imaging: comparison of 2 tracer kinetic models. *Journal of nuclear cardiology*. 2005;12:410-417.
29. Lloyd-Jones D, Adams RJ, Brown TM, Carnethon M, Dai S, De Simone G, Ferguson TB, Ford E, Furie K and Gillespie C. Heart disease and stroke statistics 2010 update. *Circulation*. 2010;121:e46-e215.
30. Mamede M, Tadamura E, Hosokawa R, Ohba M, Kubo S, Yamamuro M, Kimura T, Kita T, Saga T and Togashi K. Comparison of myocardial blood flow induced by adenosine triphosphate and dipyridamole in patients with coronary artery disease. *Annals of nuclear*

medicine. 2005;19:711-7.
31. Mettler FA, Jr., Bhargavan M, Faulkner K, Gilley DB, Gray JE, Ibbott GS, Lipoti JA, Mahesh M, McCrohan JL, Stabin MG, Thomadsen BR and Yoshizumi TT. Radiologic and nuclear medicine studies in the United States and worldwide: frequency, radiation dose, and comparison with other radiation sources--1950-2007. *Radiology*. 2009;253:520-31.
32. Muzik O, Beanlands R, Hutchins GD, Mangner TJ, Nguyen N and Schwaiger M. Validation of nitrogen-13-ammonia tracer kinetic model for quantification of myocardial blood flow using PET. *Journal of nuclear medicine: official publication, Society of Nuclear Medicine*. 1993;34:83.
33. Okada DR, Ghoshhajra BB, Blankstein R, Rocha-Filho JA, Shturman LD, Rogers IS, Bezerra HG, Sarwar A, Gewirtz H, Hoffmann U, Mamuya WS, Brady TJ and Cury RC. Direct comparison of rest and adenosine stress myocardial perfusion CT with rest and stress SPECT. *Journal of nuclear cardiology : official publication of the American Society of Nuclear Cardiology*. 2010;17:27-37.
34. Previtali M, Lanzarini L, Fetiveau R, Poll A, Ferrario M, Falcone C and Mussini A. Comparison of dobutamine stress echocardiography, dipyridamole stress echocardiography and exercise stress testing for diagnosis of coronary artery disease. *The American Journal of Cardiology*. 1993;72:865-870.
35. Rigo F, Richieri M, Pasanisi E, Cutaia V, Zanella C, Della Valentina P, Di Pede F, Raviele A and Picano E. Usefulness of coronary flow reserve over regional wall motion when added to dual-imaging dipyridamole echocardiography. *The American Journal of Cardiology*. 2003;91:269-273.
36. Rossen JD, Quillen JE, Lopez AG, Stenberg RG, Talman CL and Winniford MD. Comparison of coronary vasodilation with intravenous dipyridamole and adenosine. *J Am Coll Cardiol*. 1991;18:485-91.
37. Seret A. Will high-resolution/high-sensitivity SPECT ensure that PET is not the only survivor in nuclear medicine during the next decade? *European journal of nuclear medicine and molecular imaging*. 2009;36:533-535.
38. Sharir T, Rabinowitz B, Livschitz S, Moalem I, Baron J, Kaplinsky E and Chouraqui P. Underestimation of extent and severity of coronary artery disease by dipyridamole stress thallium-201 single-photon emission computed tomographic myocardial perfusion imaging in patients taking antianginal drugs. *Journal of the American College of Cardiology*. 1998;31:1540-1546.
39. Sharir T, Slomka PJ and Berman DS. Solid-State SPECT technology: fast and furious. *Journal of nuclear cardiology : official publication of the American Society of Nuclear Cardiology*. 2010;17:890-896.
40. Shaw LJ, Hendel R, Borges-Neto S, Lauer MS, Alazraki N, Burnette J, Krawczynska E, Cerqueira M and Maddahi J. Prognostic value of normal exercise and adenosine 99mTc-tetrofosmin SPECT imaging: results from the multicenter registry of 4,728 patients. *Journal of Nuclear Medicine*. 2003;44:134-139.
41. Smith AM, Gullberg GT and Christian PE. Experimental verification of technetium 99m-labeled teboroxime kinetic parameters in the myocardium with dynamic single-photon emission computed tomography: reproducibility, correlation to flow, and susceptibility to extravascular contamination. *Journal of nuclear cardiology*. 1996;3:130-142.
42. Tamaki N, Yonekura Y, Senda M, Yamashita K, Koide H, Saji H, Hashimoto T, Fudo T, Kambara H and Kawai C. Value and limitation of stress thallium-201 single photon emission computed tomography: comparison with nitrogen-13 ammonia positron tomography. *Journal of nuclear medicine: official publication, Society of Nuclear Medicine*. 1988;29:1181.

V. 其他國家已核准施行者，其證明文件。(Approval documents in other countries)

無 (N/A)

VI. 所需藥品或儀器設備，包括必須進口之藥品或儀器名稱、數量。(Required drugs and instruments)

1. 藥品(Drug)：

Dipyridamole

Magnevist

2. 儀器(Instruments)：

Discovery NM 530c, GE healthcare

Siemens Tim Trio 3T

VII. 行政程序，含可能引起之損害及其救濟措施、嚴重不良反應通報、是否投保等 (handling of potential adverse effect)。

單光子斷層掃瞄是非侵入性檢查，其輻射劑量約為 10 毫西弗，核磁共振檢查無輻射劑量暴露, 均是相當安全的檢查，不會有過敏的不良情形發生。注射後病患體內帶有極少量輻射，因其含量甚低，不需要特別防護。若有緊急狀況發生由計畫主持人：核子醫學科閻紫宸醫師及計畫共同主持人：核子醫學科何恭之醫師負責處理。

SPECT is a non-invasive exam that is typically safe with reasonable radiation exposure around 10 mSv. MRI is non-radioactive and most patients can safety receive such exams. After the study, residual activities within subject is very low and does not require special protection. If there are any emergent situations, the involved cardiologists and nuclear physicians will be in charge of handling.
